# Target-templated *de novo* design of macrocyclic d-/l-peptides: discovery of drug-like inhibitors of PD-1†

**DOI:** 10.1039/d1sc01031j

**Published:** 2021-03-02

**Authors:** Salvador Guardiola, Monica Varese, Xavier Roig, Macarena Sánchez-Navarro, Jesús García, Ernest Giralt

**Affiliations:** Institute for Research in Biomedicine (IRB Barcelona), The Barcelona Institute of Science and Technology Baldiri Reixac 10 08028 Barcelona Spain ernest.giralt@irbbarcelona.org; Department of Molecular Biology, Instituto de Parasitología y Biomedicina, CSIC Granada Spain; Department of Inorganic and Organic Chemistry, University of Barcelona Spain

## Abstract

Peptides are a rapidly growing class of therapeutics with various advantages over traditional small molecules, especially for targeting difficult protein–protein interactions. However, current structure-based methods are largely limited to natural peptides and are not suitable for designing bioactive cyclic topologies that go beyond natural l-amino acids. Here, we report a generalizable framework that exploits the computational power of Rosetta, in terms of large-scale backbone sampling, side-chain composition and energy scoring, to design heterochiral cyclic peptides that bind to a protein surface of interest. To showcase the applicability of our approach, we developed two new inhibitors (**PD-i3** and **PD-i6**) of programmed cell death 1 (PD-1), a key immune checkpoint in oncology. A comprehensive biophysical evaluation was performed to assess their binding to PD-1 as well as their blocking effect on the endogenous PD-1/PD-L1 interaction. Finally, NMR elucidation of their in-solution structures confirmed our *de novo* design approach.

## Abstract 2

*In silico* design of heterochiral cyclic peptides that bind to a specific surface patch on the target protein (PD-1, in this case) and disrupt protein–protein interactions.

## Graphical abstract

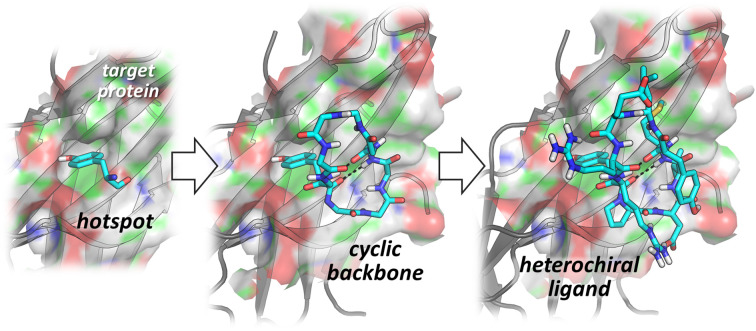

## Introduction

Protein–protein interactions (PPIs) are a relatively unexplored class of biological targets. From a drug discovery perspective, PPIs represent a challenge to conventional small-molecule drugs owing to their physicochemical properties—*i.e.* large and featureless contact patches lacking hydrophobic binding pockets.^1,2^ Due to their size and affinity, monoclonal antibodies (mAbs) are potent tools to target specific protein epitopes and block PPIs; however, there is a longstanding aspiration for smaller molecules with better pharmaceutical properties.^3–6^ Like antibodies, peptides display large and chemically diverse binding interfaces, but they show improved biodistribution and tissue penetration.^7^ In addition, they can be chemically synthesized—meaning lower costs and higher batch-to-batch reproducibility. Natural all-l peptides, however, suffer from major limitations for *in vivo* use, with regards to their high flexibility—resulting in a largely disordered conformation, and poor resistance to proteolytic degradation. To overcome these problems, cyclic peptides, especially those including d-amino acids, have emerged as a distinct class of therapeutics with suitable drug-like properties: since they are endowed with high structural rigidity, biological stability and favorable pharmacokinetics.^8,9^

Computational modelling tools for peptides have greatly evolved in recent years; however, most methods are trained on sequence-structure data of large natural proteins and thus fail to be efficient for predicting the structures of small peptides with unnatural modifications.^10,11^ Likewise, most structure-based design efforts made with peptides have focused on mimicking linear epitopes in one of the partners of the PPI, typically featuring well-defined structural motifs such as an α-helix binding to an elongated cleft on the other protein.^12–14^ However, many PPIs are mediated by non-linear epitopes for which some degree of structural plasticity such as side-chain and backbone rearrangement occur upon complex formation.^15^ In this context, designing cyclic peptides from scratch with highly pre-organized structures to bind their targets is a challenging computational task due to the need to simultaneously optimize two independent factors: (i) backbone geometries with distinctive energy minimums that stabilize the desired peptide fold;^16^ and (ii) side-chain composition—including d-amino acids—that maximizes surface complementarity and free energy of binding to the target. The Rosetta modelling suite has been successfully applied to the *de novo* design of functional proteins with a myriad of applications.^17,18^ Recently, Rosetta has incorporated features that are not derived from natural proteins, such as the GenKIC method^19^ for sampling non-standard cyclic peptide chemistries. Here, we have exploited this and other structure-based tools to design mixed-chirality cyclic peptides, in the presence of a target protein as template.

To showcase the potential of our approach, we chose as model system the PD-1/PD-L1 interaction due to its relevance in cancer immunotherapy. Programmed cell death 1 (PD-1) is a cell membrane receptor expressed in lymphocytes and other immune cells. It has two naturally occurring ligands, PD-L1 and PD-L2, which are normally present in a variety of endogenous cell populations.^20^ However, some tumor types are able to overexpress these ligands as a mechanism to evade immune surveillance.^21^ In recent years, therapeutic targeting of PD-1 with mAbs has brought about a major breakthrough in the fight against melanoma, lung cancer, and other types of cancer.^22^ However, from a drug discovery point of view, the PD-1/PD-L1 interaction continues to be a challenging PPI that has been successfully drugged only using mAbs.^23^ Thus, here we aimed to design macrocyclic constrained peptides, formed by both l- and d-residues, that work as ligand decoys by targeting PD-1 and hence preventing the binding of either PD-L1 or PD-L2.

## Results

### Hotspot selection and *in silico* backbone sampling for peptide design

**Fig. 1:**
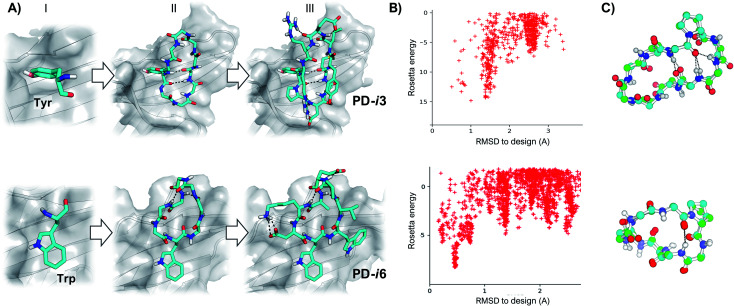
Design of peptide macrocycles targeting PD-1. (A) Schematic design protocol for **PD-i3** (top) and **PD-i6** (bottom). Starting from a fixed hotspot residue (I), a cyclic poly-Gly backbone is built and thousands of conformations are sampled with kinematic closure (II); ultimately, low-energy conformations are selected and side-chain designed (III) to maximize favorable contacts with the target protein. (B) Energy landscape calculations show convergence towards low-energy structures that are close to the design. (C) Superposition of the lowest-energy energy structure (in green) with the target-templated design (in blue). For the sake of clarity, only the backbone and Pro residues are shown.

As shown by X-ray crystallography, PD-1 binds its partner proteins (PD-L1 and PD-L2) through a large and flat β-sheet interface that buries a total surface area of 1970 Å^2^.^24^ Comparison of the apo (PDB ID: 3RRQ) and PD-L1-bound (PDB ID: 4ZQK) structures of PD-1 reveals that binding to PD-L1 induces an important rearrangement of the flexible CC′ β-sheet region of PD-1 (residues M70-D77) (Fig. S1†).^25^ This backbone reordering opens a transient hydrophobic pocket that accommodates the aromatic side chain of Y123 in PD-L1 and enables key interactions with adjacent residues. Importantly, Y123 is conserved in all known PD-L1 and PD-L2 sequences, and it has been further confirmed as a main hotspot by several studies^24,26^ and prediction algorithms (Fig. S2†). Hence, we selected Y123 as an anchor point on which to build our cyclic peptides. From this anchor, while keeping the target PD-1 as template throughout the design process, backbones were generated by appending 4–5 Gly residues to the N- and C-termini of Y123, respectively (Fig. 1). Since Gly is the only naturally occurring amino acid that explores both sides of the Ramachandran plot, poly-Gly backbones are a suitable model for the design of heterochiral topologies. On the other hand, 8–10 residue peptides yield a convenient balance between a large contact surface—thus binding energy—and constrained flexibility (larger peptides typically result in more than one conformation in solution).

Next, cyclic poly-Gly conformations were sampled using the robotics-based kinematic closure (GenKIC) algorithm.^19^ This method calculates tens of thousands of mechanically accessible backbone conformers, now in the presence of the target protein, while randomizing *φ* and *ψ* angles biased by all preferred l- and d-regions in the Ramachandran plot. High-energy structures (*E* > 0) were prematurely discarded due to steric clashes with PD-1; also, solutions with no or very few intramolecular backbone hydrogen bonds were filtered. Monte Carlo sampling was then performed to introduce, minimize and select low-energy side-chain rotamers for all residues in the peptide except Y123. In this process, residues with dihedral angle *φ* < 0 were allotted to l-amino acids, while positions with *φ* > 0 were designed as d-amino acids. Gly and Ala were penalized to enhance chemical diversity, and l- or d-Pro were favored to restrain the flexibility of the newly generated backbones (Fig. 1). Finally, low-energy structures were ranked based on their free energy of binding, interface buried solvent-accessible area, shape complementarity and total number of inter- and intra-molecular hydrogen bonds—which favor target affinity and peptide folding, respectively.^27^ After visual inspection, the top-scoring poses were subjected to independent energy landscape calculations in order to assess the degree of structural pre-organization (Fig. 1). Only a few solutions revealed folding funnels with low-energy structures that were close to the designed conformation (root-mean-square deviation, RMSD < 1 Å), from which two peptides (**PD-i1-2**) were chosen for further experimental evaluation (Fig. S3†).

### New hotspot geometries yield alternative designs

In these initial attempts, however, although we identified low-energy backbones that matched the designs, we were not able to recapitulate the unusual rotameric dihedrals (*χ*_1_ = 59°, *χ*_2_ = 86°) that Tyr123 adopts when binding to PD-1 (Fig. S3†). To address this issue, we explored more frequent side-chain geometries for the Tyr hotspot, and selected the lowest-energy rotamer that did not produce backbone clashes with the PD-1 interface (*χ*_1_ = 180°, *χ*_2_ = 80°). From this Tyr hotspot, an analogous design protocol to the one described above was followed to generate heterochiral peptides **PD-i3-4**.

As a third starting point for our designs and given that the PD-1 binding interface shows substantial flexibility in the absence of ligands,^25^ we sought to explore the structure of human PD-1 in its unbound state (PDB ID: 3RRQ). In this case, the FTMap algorithm^28^ was used to probe for “sticky” binding sites on the protein surface. Surprisingly, despite the CC′ loop now being in its closed conformation, the same PD-L1 interface site still ranked first in terms of “druggability”. From the chemical probes sampled with FTMap, we hypothesized that this large and relatively flat region could accommodate large aromatic rings the size of a Trp side chain (Fig. S4†). Thus, we docked this amino acid on the protein and used it as anchor residue for assembling cyclic peptides against apo PD-1 (**PD-i5-7**, Fig. 1 and S5†).

### Efficient peptide synthesis and binding assays against human PD-1

**Fig. 2:**
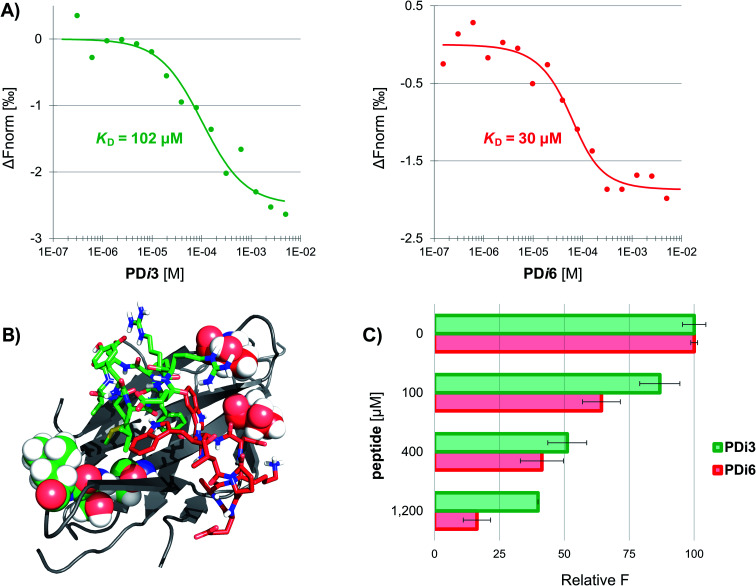
The designed macrocycles bind to PD-1 and disrupt the interaction with PD-L1. (A) Normalized MST signals for the titration of human PD-1 with **PD-i3** (left) and **PD-i6** (right). Measurements performed in triplicate. (B) NMR mapping of the interaction sites of **PD-i3** and **PD-i6** (in green and red sticks, respectively). PD-1 residues that undergo significant changes in signal intensity are shown as CPK representation and are color-coded according to the ligand. (C) PD-1/PD-L1 AlphaScreen inhibition assay. Measurements performed in triplicate.

Seven *de novo* designed cyclic peptides were in total selected for experimental characterization (**PD-i1-7**). To this end, peptide chains were elongated on a Wang-linker modified polystyrene resin (anchoring through the Asp side-chain carboxylic acid). This chemical strategy enabled the head-to-tail cyclisation to be readily performed on-resin, and a final deprotection/cleavage step yielded the already cyclized final peptide (Scheme S1†). To test for their bioactivity, the extracellular domain of human PD-1 was recombinantly expressed in *E. coli* and refolded from inclusion bodies. The purified protein was then immobilized by free amine coupling on a CM5 sensor chip surface, and surface plasmon resonance (SPR) measurements were performed. Two of the seven designs (**PD-i3** and **PD-i6**) showed distinct concentration-dependent SPR responses, which were specific for the PD-1-functionalised channel, while the other ligands showed little or no response (Fig. S6†). Due to fast kinetics and a lack of signal saturation (even when the endogenous PD-L1 was assayed),^29^ we decided to explore alternative techniques for more accurate dissociation constant (*K*_D_) calculation. In particular, we selected microscale thermophoresis (MST) as it has been validated with this system^30^ and allows the quantification of interactions in solution, which mimics physiological conditions. To this end, PD-1 was first fluorescently labelled and then titrated with increasing amounts of **PD-i3** and **PD-i6.** A reproducible shift in the thermophoresis signal was observed in both cases, yielding affinity values in the low-to-mid micromolar range—in the same order of magnitude as the PD-1/PD-L1 interaction (Fig. 2A and S7†).^30^ These results were independently confirmed by isothermal titration calorimetry experiments (Fig. S8†). Finally, to probe for the specificity of the binding interactions, we mutated some of the critical amino acids in **PD-i6**—the most active binder—and performed MST binding affinity measurements. Mutation of l-Trp, which serves as PD-1 anchor for the *in silico* design process, largely abolished binding to the target (Fig. S9A†). Likewise, replacement of the turn-inducing d-Pro residue for d-Ala—or even flipping its chirality to l-Pro—led in both cases to losses in binding affinity of >100-fold (Fig. S9B–C†), thus showing the functional importance of these peptide residues, in line with the predicted computational design.

### **PD-i3** and **PD-i6** target the PD-1/PD-L1 interface and disrupt the interaction

Next, we examined the capacity of **PD-i3** and **PD-i6** to inhibit the PD-1/PD-L1 interaction in a fluorescence-transfer AlphaScreen assay, which mimics the protein settings found in cell membranes *in vivo.*^31^ In this assay, a concentration-dependent inhibition of the interaction was observed for both cyclic peptides; this effect was nevertheless stronger for **PD-i6**, in agreement with their relative binding affinities to PD-1 (Fig. 2C). To further elucidate the interaction site of **PD-i3** and **PD-i6**, we expressed the extracellular domain of PD-1 uniformly labelled in ^15^N and performed high-resolution NMR spectroscopy assays. In our hands, however, the ^1^H, ^15^N HSQC spectra of free PD-1 showed the absence of some amide signals, especially from residues located on the CC′ loop—something that typically occurs in flexible regions of proteins (Fig. S10†). Despite this issue, the addition of either **PD-i3** or **PD-i6** to the protein led to significant changes in signal intensity (>20%) in residues located adjacent to the CC′ loop, which correlate with the expected binding sites of each peptide (Fig. 2B and S11†) and is consistent with the PPI-disrupting activity observed in the AlphaScreen assay.

### Solution structures of **PD-i3** and **PD-i6** validate the designs

**Fig. 3:**
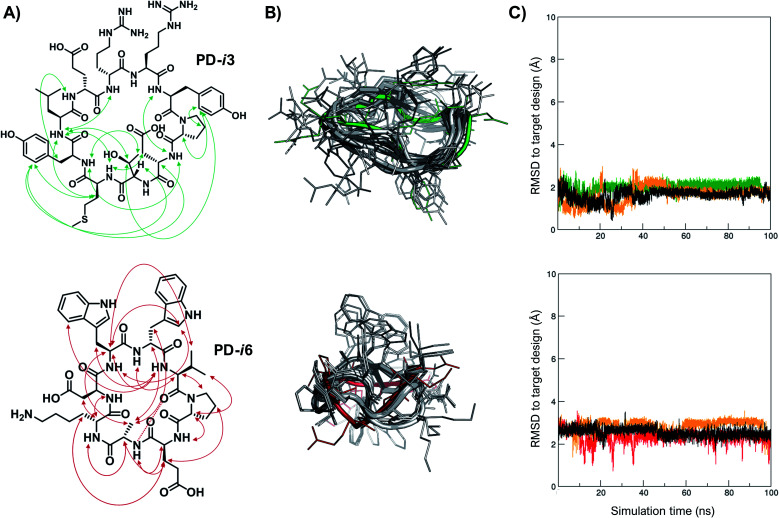
Experimental structures match the designs. (A) Observed NOEs for **PD-i3** (top) and **PD-i6** (bottom). (B) Overlay of the designed model (green and red) with the ensemble of the 10 lowest-energy structures (in grey) for each peptide in water. (C) RMSD to the designed conformation during unrestrained MD trajectories (3 × 100 ns), starting from the lowest-energy NMR structure.

Finally, in order to experimentally validate the results of our structure-based *de novo* design method, we performed a comprehensive NMR characterization of the structures of **PD-i3** and **PD-i6** in solution. Both peptides showed well-dispersed ^1^H monodimensional spectra with a single set of ^1^HN backbone signals, indicative of a single conformation (Fig. S12†). We further collected short-, medium- and long-range nuclear Overhauser effect (NOE) correlations, which were set as restraints for simulated annealing calculations using Xplor-NIH (Fig. 3A and ESI†).^32^ A total of 1000 low-energy structures were obtained that satisfied the observed NOE distance constraints (Fig. 3B). From the lowest-energy conformation of this NMR ensemble, we launched unrestrained molecular dynamics (MD) simulations and compared the trajectories to the initially designed structures. For **PD-i6**, the design aligns to the NMR ensemble with a root-mean-square deviation (RMSD, for all heavy atoms) of 1.7–2.2 Å (depending on the ensemble structure used for comparison) and remains stable during the MD simulation in each of the 3 replicas (Fig. 3C). The cyclic backbone geometry is well-preserved, while the side chains show higher conformational freedom—a typical behavior of small peptides lacking hydrophobic core packing. For **PD-i3**, the NMR structure is nearly identical to the target design (all-atom RSMD of 1.1 Å), a notable result given the larger size of this peptide. Importantly, free MD simulations of **PD-i3** revealed minimal backbone fluctuations and a high preservation of the designed bioactive conformation (RMSD ≤ 2 Å), thereby validating our initial PD-1-templated computational design approach.

## Discussion and conclusions

Computational tools for predicting the three-dimensional structure of peptides have greatly advanced in recent years. However, there are still major hurdles on the way to more robust and versatile prediction methods that can also be applied to the inverse problem, namely the design of amino acid sequences that stabilize, for instance, a particular binding-competent peptide conformation. On the one hand, a relatively small number of peptide structures, compared to proteins, have been experimentally characterized and are available in the PDB. Even fewer structures correspond to unnatural peptides and peptidomimetics, although this number has been steadily increasing. On the other hand, small peptides have relatively flat energy landscapes, in contrast to most proteins—intrinsically disordered proteins being a notable exception—and sample an ensemble of conformations in solution. In this scenario, protein-based methods that predict local features, such as secondary structural motifs or backbone angles, on the basis of sequence similarity to previously determined structures, have largely failed for peptides.^33^ Fortunately, more recent approaches based on sequence-independent fragment-free sampling, such as robotics-inspired kinematic closure moves,^19^ are more efficient to model small peptide loops and segments regardless of the amino acid chirality and can tolerate artificial cyclic constraints.^34^

In this work, we have applied Rosetta modelling tools to design bioactive peptides that complementarily bind to a specific surface patch on the target protein. As desirable features that we considered, drug-like peptides should incorporate non-proteogenic amino acids to improve their metabolic stability *in vivo*. In addition, they should be constrained into a functional conformation that makes target engagement easier by minimizing entropic loss caused by binding. This structural rigidification can be achieved by large-scale sampling of cyclic backbones, using the kinematic closure (GenKIC) method, selection of low-energy dihedrals that favor the desired peptide fold, and introduction of Pro residues to stabilize kink points in the sequence. Finally, the side chains are designed to maximize binding free energy and surface complementarity with the target.

As a case study, we chose the PD-1 cellular receptor since it is an extracellular target and it plays a key role in the evasion of immune surveillance by cancer cells. The target has been clinically validated using mAbs, which have shown outstanding results in patients. However, no small molecules or peptides are available yet. PD-1 is a challenging target for structure-based design because it presents a flat and featureless binding interface—formed by discontinuous peptide epitopes enriched in β-sheet content—and shows significant backbone and side-chain plasticity, which is manifest upon complex formation with its partners PD-L1/L2 (Fig. S1†). Aware that modelling artifacts are likely to occur during the design process, we selected various input structures of the target as starting points for the design. In fact, our first attempts using the crystal structure of the PD-1/PD-L1 complex as input resulted in peptides with no or little activity (**PD-i1**-**4**). This might be due to the substantial remodeling of the PD-1 binding interface, which opens a transient binding site that is not found in free-state PD-1, a result that is in agreement with previous negative results using fragments.^24^ In addition to the input structure used for the target, another relevant point emerging from this study is the importance of selecting adequate geometries for anchoring the hotspot to the target—notably the side-chain rotameric angles—since it is the only peptide segment that is fixed throughout the design process.

In conclusion, we have designed structurally constrained heterochiral cyclic peptides with three-dimensional shapes and chemical functionalities that are complementary to a specific protein–protein interface of interest. Two of our designed peptides (**PD-i3** and **PD-i6**) showed mid-micromolar binding affinity to the target PD-1 and outcompeted endogenous PD-1 ligands for disrupting the PD-1/PD-L1 interaction. Importantly, their experimental structures, as determined by NMR, were in close agreement with the predicted computational designs, thus validating our design methodology. Given the growing interest in peptides as therapeutic modality, this *in silico* workflow could be interfaced with powerful experimental technologies, such as phage display^35,36^ and synthetic libraries^37^ as strategies to generate new drug-like peptide candidates from scratch against other challenging targets.

## Materials and methods

For detailed procedures and resources, please refer to the ESI† section.

### Computational design of macrocyclic peptide binders

Peptide *de novo* design was divided into 3 main steps: hotspot selection, cyclic backbone sampling and sequence design. For hotspot selection, peptides **PD-i1** to **PD-i4** were designed on the basis of the X-ray structure of the PD-1/PD-L1 complex (PDB ID: 4ZQK). Following *in silico* Ala scan results (Fig. S2†), as well as previous observations;^24,26^ Y123 was selected as main hotspot residue of the interaction. On the other hand, as a different starting point for peptides **PD-i5** to **PD-i7**, the apo-PD-1 form of the protein (PDB ID: 3RRQ) was screened for hydrophobic surface patches using the FTMap algorithm.^28^ The first-scoring site, located on the PD-1/PD-L1 interface, was selected to dock l-Trp as hotspot, with its flat aromatic side chain optimally docked on the protein binding site. Then, from each respective hotspot, cyclic poly-Gly backbones of 8, 9 and 10 residues were built, in the presence of the target protein structure. Exhaustive conformational sampling was performed using the GenKIC method^19^ implemented in the Rosetta modeling suite, with restrictions for the main ABEGO bins of the Ramachandran space. Solutions were filtered based on the Rosetta score function, as well as on the number of backbone hydrogen bonds. Finally, sequence design was performed using the FastDesign protocol, while keeping the main hotspot fixed. Fast design alternates sidechain rotamer optimization and gradient-descent-based energy. Residues with backbone dihedral *φ* < 0 were designed as l-amino acids, while positions with *φ* > 0 became d-amino acids. Low-energy structures were ranked following several metrics to enhance target binding affinity, such as free energy of binding, interface buried solvent-accessible area, shape complementarity and total number of inter- and intra-molecular hydrogen bonds. A sample Rosetta XML script comprising all the aforementioned steps is provided in the ESI.†

### Conformational energy landscape calculations

Due to the reduced size of the peptides and the large number of unnatural d-amino acids, we employed the simple_cycpep_predict algorithm,^19^ which does not rely on PDB-derived fragments, to assess the level of structural preorganization of the peptides. Designs showing poor sampling near the designed bioactive conformation were discarded.

### Peptide synthesis

Peptides were synthesized by automated solid phase peptide synthesis (SPPS) on a microwave peptide synthesizer. Side-chain anchoring on a Wang polystyrene solid support and on-resin intramolecular cyclization were the key steps to afford in a facile manner the designed cyclic peptides (see the ESI† for more details).

### Recombinant expression of human PD-1

The extracellular domain of human PD-1 was recombinantly expressed in *E. coli* BL21(DE3) in the form of inclusion bodies, by adapting a previously described procedure.^24^

### Bioactivity experimental assays

The binding of peptides to **PD-i1** to **PD-i7** was comprehensively studied using an array of biophysical techniques such as surface plasmon resonance (SPR), microscale thermophoresis (MST), isothermal calorimetry (ITC), AlphaScreen®, and high-field nuclear magnetic resonance (NMR). Full experimental details for each technique are provided in the ESI.†

### Structural determination

The solution structures of **PD-i3** and **PD-i6** were solved by the combined analysis of 2D homo-(TOCSY, NOESY) and natural-abundance hetero-nuclear ^1^H–^13^C HSQC NMR experiments, recorded at 5 °C, pH 6.4. From these data, structural ensembles of the 10 lowest-energy conformations were calculated by simulated annealing calculations with the Xplor-NIH software. Coordinates for these structures were deposited at the Protein Data Bank under accession codes 6TVJ (for **PD-i3**) and 6TT6 (for **PD-i6**).

## Author contributions

S. G. wrote the manuscript, designed and performed the research, supervised the experiments, and analyzed the data; M. V. designed the research and analyzed the data; X. R. performed the research; M. S. N. performed revision experiments and analyzed the data; J. G. supervised the experiments and analyzed the data; E. G. designed the research, supervised the experiments and revised the manuscript. All authors commented on the manuscript.

## Conflicts of interest

The authors declare no competing interests.

## Supplementary Material

SC-012-D1SC01031J-s001
