# Supplementary material for: Target-templated de novo design of macrocyclic d-/l-peptides: discovery of drug-like inhibitors of PD-1
Source: Chem Sci. 2021 Mar 2;12(14):5164–70. doi: 10.1039/d1sc01031j (PMC8179567; doi:10.1039/d1sc01031j)
Supplement: SC-012-D1SC01031J-s001 [file SC-012-D1SC01031J-s001.pdf]

Electronic Supplementary Material (ESI) for Chemical Science.
This journal is © The Royal Society of Chemistry 2021

Target-templated *de novo* design of macrocyclic D-/L-peptides: discovery of drug-like inhibitors of PD-1

Salvador Guardiola,[1*] Monica Varese,[1] Xavier Roig,[1]
Macarena Sánchez-Navarro,[2] Jesús García,[1] and Ernest Giralt[1,3*]

[1]Institute for Research in Biomedicine (IRB Barcelona), The Barcelona Institute of Science and Technology, Baldiri Reixac 10, 08028 Barcelona, Spain
[2]Department of Molecular Biology, Instituto de Parasitología y Biomedicina, CSIC, Granada, Spain
[3]Department of Inorganic and Organic Chemistry, University of Barcelona, Spain
*Correspondence: ernest.giralt@irbbarcelona.org

SUPORTING INFORMATION

MATERIALS AND METHODS 2
- Computational design of cyclic heterochiral peptides 2
- Energy landscape calculation of peptide macrocycles 8
- General conditions for peptide synthesis 8
- Synthesis of cyclic peptides **PD-*i*1-7** 8
- Recombinant expression of human PD-1 9
- Surface plasmon resonance (SPR) 9
- Microscale thermophoresis (MST) 10
- Isothermal titration calorimetry (ITC) 10
- NMR spectroscopy 10
- NMR structural determination 10
- Molecular dynamics (MD) 11

SUPPORTING FIGURES 12
- Figure S1 12
- Figure S2 13
- Figure S3 14
- Figure S4 15
- Figure S5 16
- Figure S6 17
- Figure S7 18
- Figure S8 19
- Figure S9 20
- Figure S10 21
- Figure S11 22
- Figure S12 23

SUPPORTING DATA 24
- UPLC-MS of peptides **PD-*i*1-7** 24
- SEC-MALS and MS of recombinantly expressed human PD-1 28
- NMR assignment of **PD-*i*3** and **PD-*i*6** 29
- NOE distance restraints used for structure calculation 31

BIBLIOGRAPHY 32

Materials and methods

Peptide and protein modelling were performed using Rosetta v2018.26 and Amber14. All amino acids, resins, solvents and reagents were purchased from Bachem AG (Bubendorf, Switzerland), Iris Biotech (Marktredwitz, Germany), Scharlab (Barcelona, Spain), Sigma-Aldrich (Milwaukee, WI), GL Biochem Shangai Ltd (Shangai, China), and Fluka Chemika (Buchs, Switzerland). Reagents for microscale thermophoresis (MST) and AlphaScreen were purchased from NanoTemper Technologies GmbH (München, Germany) and PerkinElmer (Waltham, MA). General material (sterile and not sterile) was acquired from Sharlab (Barcelona, Spain) and Deltalab.

Computational design of cyclic heterochiral peptides

Starting from a PDB file containing the coordinates of the extracellular domain of PD-1 (chain A, residues 1-106) bound to an anchoring hotspot residue (chain B), a sample Rosetta XML script used for generating 10-mer heterochiral cyclic peptides is shown below:

```
<ROSETTASCRIPTS>

    <SCOREFXNS>

        #general score function
        <ScoreFunction name="beta" weights="beta_nov16"/>

        #composition score function for designing the side chains
        <ScoreFunction name="beta_comp" weights="beta_nov16">
            <Reweight scoretype="coordinate_constraint" weight="1"/>
            <Reweight scoretype="atom_pair_constraint" weight="1"/>
            <Reweight scoretype="dihedral_constraint" weight="1"/>
            <Reweight scoretype="angle_constraint" weight="1"/>
            <Reweight scoretype="aa_composition" weight="1.0"/>
        </ScoreFunction>

        #score function for checking backbone H-bonds
        <ScoreFunction name="high_hbond" weights="beta_nov16">
            <Reweight scoretype="coordinate_constraint" weight="1"/>
            <Reweight scoretype="atom_pair_constraint" weight="1"/>
            <Reweight scoretype="dihedral_constraint" weight="1"/>
            <Reweight scoretype="angle_constraint" weight="1"/>
            <Reweight scoretype="hbond_lr_bb" weight="11.7"/>
            <Reweight scoretype="hbond_sr_bb" weight="11.7"/>
        </ScoreFunction>

    </SCOREFXNS>

    <RESIDUE_SELECTORS>

        #selecting anchor hotspot
        <Index name="TYR_sel" resnums="111"/>

        #selecting the chains
        <Chain name="peptide" chains="B"/>
        <Chain name="PD1" chains="A"/>

        #selecting D and L amino acids
        <Phi name="posPhi" select_positive_phi="true" />
        <And name="posPhi_pep" selectors="peptide,posPhi"/>
        <Phi name="negPhi" select_positive_phi="false" />
        <And name="negPhi_pep" selectors="peptide,negPhi"/>
```

```
        #selecting interface of chainB close within 8.0 A of chainA
        <Neighborhood name="interface_chA" selector="peptide" distance="8.0"/>

        #selecting interface of chainA close within 8.0 A of chainB
        <Neighborhood name="interface_chB" selector="PD1" distance="8.0"/>

        #selecting intersection of chain A and Chain B interfaces
        <And name="AB_interface" selectors="interface_chA,interface_chB"/>

        #select residues for backbone H-bond filter
        <Index name="hbonds_to_107" resnums="109,110,111,112,113,114,115"/>
        <Index name="hbonds_to_108" resnums="110,111,112,113,114,115,116"/>
        <Index name="hbonds_to_109" resnums="107,111,112,113,114,115,116"/>
        <Index name="hbonds_to_110" resnums="107,108,112,113,114,115,116"/>
        <Index name="hbonds_to_111" resnums="107,108,109,113,114,115,116"/>
        <Index name="hbonds_to_112" resnums="107,108,109,110,114,115,116"/>
        <Index name="hbonds_to_113" resnums="107,108,109,110,111,115,116"/>
        <Index name="hbonds_to_114" resnums="107,108,109,110,111,112,116"/>
        <Index name="hbonds_to_115" resnums="107,108,109,110,111,112,113"/>
        <Index name="hbonds_to_116" resnums="108,109,110,111,112,113,114"/>

    </RESIDUE_SELECTORS>

    <TASKOPERATIONS>

        #resfiles for D and L residues
        <ReadResfile name="l_res" filename="l_res.txt" selector="negPhi_pep"/>
        <ReadResfile name="d_res" filename="d_res.txt" selector="posPhi_pep"/>

        #only minimize protein residues that are on the binding interface, do
not design anchor hotspot
        <RestrictChainToRepacking name="only_repack_chain" chain="1"/>
        <OperateOnResidueSubset name="no_design_TYR" selector="TYR_sel">
            <RestrictToRepackingRLT/>
        </OperateOnResidueSubset>

        <OperateOnResidueSubset name="no_repack_except_interface">
            <And>
                <Chain chains="1"/>
                <Not>
                    <Neighborhood distance="8.0">
                        <Chain chains="2"/>
                    </Neighborhood>
                </Not>
            </And>
            <PreventRepackingRLT />
        </OperateOnResidueSubset>

        <OperateOnResidueSubset name="restrict_to_interface"
selector="AB_interface">
            <RestrictToRepackingRLT/>
        </OperateOnResidueSubset>

        #do not introduce Gly or Cys on the peptide
       <DisallowIfNonnative name="no_cys" disallow_aas="CG"/>


       #prevents rotamers of aromatic residues with rare dihedrals
        <LimitAromaChi2 name="limchi2"/>

       #include current rotamers
        <IncludeCurrent name="current"/>
```

```
    </TASKOPERATIONS>

    <FILTERS>
        #limit the number of Gly
       <ResidueCount name="gly_count" max_residue_count="2"
min_residue_count="0" residue_types="GLY" count_as_percentage="0"
confidence="0" />

       #total energy score
        <ScoreType name="score" scorefxn="beta" score_type="total_score"
threshold="-650" confidence="0" />

        #filter to discard very bad structures after genKIC
       <ScoreType name="score_low" scorefxn="beta" score_type="total_score"
threshold="0"/>

        #interface shape complementarity
        <ShapeComplementarity name="sc_filter" verbose="0" min_sc="0.6"
write_int_area="1" jump="1" confidence="1"/>

        #computes ddG with and without minimizing
        <Ddg name="ddg"  threshold="-1" jump="1" repeats="5" repack="1"
confidence="1" scorefxn="beta"/>
        <Ddg name="ddg_norepack"  threshold="-1" jump="1" repeats="1"
repack="0" confidence="1" scorefxn="beta"/>

        #computes the interface sasa (total, hydrophobic, and polar)
        <Sasa name="interface_buried_sasa" confidence="0"/>
        <Sasa name="interface_hydrophobic_sasa" confidence="0"
hydrophobic="True"/>
        <Sasa name="interface_polar_sasa" confidence="0" polar="True"/>

        #calculates number of burried unsatisfied interface H-bond
donors/aceptors (side-chain and mainchain)
       <BuriedUnsatHbonds name="new_buns_sc_heavy" use_reporter_behavior="true"
report_sc_heavy_atom_unsats="true" scorefxn="beta"
residue_surface_cutoff="15.0" ignore_surface_res="true"
print_out_info_to_pdb="true" confidence="0" residue_selector="AB_interface"/>

        <BuriedUnsatHbonds name="new_buns_bb_heavy"
use_reporter_behavior="true" report_bb_heavy_atom_unsats="true"
scorefxn="beta" residue_surface_cutoff="15.0" ignore_surface_res="true"
print_out_info_to_pdb="true" confidence="0" residue_selector="AB_interface"/>

       #computes the number of mainchain H-bonds within the peptide, threshold
can be higher or lower
        <HbondsToResidue name="hbonds1" partners="0" energy_cutoff="-0.25"
backbone="1" bb_bb="1" sidechain="0" residue="107"
residue_selector="hbonds_to_107"/>
        <HbondsToResidue name="hbonds2" partners="0" energy_cutoff="-0.25"
backbone="1" bb_bb="1" sidechain="0" residue="108"
residue_selector="hbonds_to_108"/>
        <HbondsToResidue name="hbonds3" partners="0" energy_cutoff="-0.25"
backbone="1" bb_bb="1" sidechain="0" residue="109"
residue_selector="hbonds_to_109"/>
        <HbondsToResidue name="hbonds4" partners="0" energy_cutoff="-0.25"
backbone="1" bb_bb="1" sidechain="0" residue="110"
residue_selector="hbonds_to_110"/>
        <HbondsToResidue name="hbonds5" partners="0" energy_cutoff="-0.25"
backbone="1" bb_bb="1" sidechain="0" residue="111"
residue_selector="hbonds_to_111"/>
```

```
        <HbondsToResidue name="hbonds6" partners="0" energy_cutoff="-0.25"
backbone="1" bb_bb="1" sidechain="0" residue="112"
residue_selector="hbonds_to_112"/>
        <HbondsToResidue name="hbonds7" partners="0" energy_cutoff="-0.25"
backbone="1" bb_bb="1" sidechain="0" residue="113"
residue_selector="hbonds_to_113"/>
        <HbondsToResidue name="hbonds8" partners="0" energy_cutoff="-0.25"
backbone="1" bb_bb="1" sidechain="0" residue="114"
residue_selector="hbonds_to_114"/>
        <HbondsToResidue name="hbonds9" partners="0" energy_cutoff="-0.25"
backbone="1" bb_bb="1" sidechain="0" residue="115"
residue_selector="hbonds_to_115"/>
        <HbondsToResidue name="hbonds10" partners="0" energy_cutoff="-0.25"
backbone="1" bb_bb="1" sidechain="0" residue="116"
residue_selector="hbonds_to_116"/>

        <CombinedValue name="total_hbonds" threshold="-3.0">
            <Add filter_name="hbonds1" factor="-0.5"/>
            <Add filter_name="hbonds2" factor="-0.5"/>
            <Add filter_name="hbonds3" factor="-0.5"/>
            <Add filter_name="hbonds4" factor="-0.5"/>
            <Add filter_name="hbonds5" factor="-0.5"/>
            <Add filter_name="hbonds6" factor="-0.5"/>
            <Add filter_name="hbonds7" factor="-0.5"/>
            <Add filter_name="hbonds8" factor="-0.5"/>
            <Add filter_name="hbonds9" factor="-0.5"/>
            <Add filter_name="hbonds10" factor="-0.5"/>
        </CombinedValue>

    </FILTERS>

    <MOVERS>

        #extend the peptide chain by adding Gly residues to the anchor hotspot
        <PeptideStubMover name="extend" reset="0">
            <Prepend resname="GLY" anchor_rsd="107" repeat="4"/>
            <Append resname="GLY" repeat="5"/>
        </PeptideStubMover>

        #set omega dihedrals to 180°
        <SetTorsion name="set_torsion">
            <Torsion residue="107,108,109,110,111,112,113,114,115,116"
torsion_name="omega" angle="180"/>
        </SetTorsion>

        #cyclize head-to-tail peptide
        <PeptideCyclizeMover name="cyclize" residue_selector="peptide"/>

        #pre-selection criteria for genKIC
        <ParsedProtocol name="preselection_pp">
            <Add filter="total_hbonds"/>
            <Add filter="score_low"/>
        </ParsedProtocol>

        #genKIC mover, takes into account the number of H-bonds to favour
structurally constrained structures
        <GeneralizedKIC name="genkic" closure_attempts="1000"
stop_when_n_solutions_found="1" selector="lowest_energy_selector"
selector_scorefunction="high_hbond" pre_selection_mover="preselection_pp">

            <AddResidue res_index="112"/>
            <AddResidue res_index="113"/>
            <AddResidue res_index="114"/>
```

```
            <AddResidue res_index="115"/>
            <AddResidue res_index="116"/>
            <AddResidue res_index="107"/>
            <AddResidue res_index="108"/>
            <AddResidue res_index="109"/>
            <AddResidue res_index="110"/>

            <CloseBond res1="116" atom1="C" res2="107" atom2="N"
bondlength="1.32" angle1="114" angle2="123" torsion="180"/>
            <SetPivots res1="112" res2="115" res3="109" atom1="CA" atom2="CA"
atom3="CA"/>

           #backbone dihedrals are randomized biased by the Ramachandran plot
            <AddPerturber effect="randomize_alpha_backbone_by_rama"
custom_rama_table="flat_symm_dl_aa_ramatable">
                <AddResidue index="113"/>
                <AddResidue index="114"/>
                <AddResidue index="116"/>
                <AddResidue index="107"/>
                <AddResidue index="108"/>
                <AddResidue index="110"/>
            </AddPerturber>

            #discard solutions with obvious clashes
           <AddFilter type="loop_bump_check"/>
        </GeneralizedKIC>

        #autoamtically set a suitable atom tree
        <AtomTree name="docking_tree" docking_ft="1"/>

        #small perturbations on peptide to allow sampling of other minima
        <RollMover name="roll" chain="1" random_roll="true"/>
        <GenericMonteCarlo name="roll_gmc" mover_name="roll" filter_name="ddg"
trials="5"/>

        #side-chain composition file to limit Ala and favor Pro residues
        <AddCompositionConstraintMover name="limitAla"
filename="limit_ALA.comp" selector="peptide"/>
        <AddCompositionConstraintMover name="bonusPro"
filename="bonus_PRO.comp" selector="peptide"/>

        #side-chain peptide design
        <FastDesign name="design"
task_operations="d_res,l_res,only_repack_chain,no_repack_except_interface,no_d
esign_TYR,no_cys,limchi2,current" scorefxn="beta_comp" repeats="5"
ramp_down_constraints="false">
            <MoveMap name="specifics">
                <Jump number="1" setting="0" />
                <Chain number="1" chi="0" bb="0"/>
            </MoveMap>
        </FastDesign>

    </MOVERS>

    <APPLY_TO_POSE>
    </APPLY_TO_POSE>

    <PROTOCOLS>
        <Add mover="extend"/>
        <Add mover="set_torsion"/>
        <Add mover="cyclize"/>
        <Add mover="genkic"/>
        <Add mover="docking_tree"/>
```

```
        <Add mover="cyclize"/>
        <Add mover="limitAla" />
        <Add mover="bonusPro" />
        <Add mover="design"/>
        <Add mover="roll_gmc"/>
        <Add mover="cyclize"/>
        <Add filter="score"/>
        <Add filter="sc_filter"/>
        <Add filter="ddg"/>
        <Add filter="ddg_norepack"/>
        <Add filter="interface_buried_sasa"/>
        <Add filter="interface_hydrophobic_sasa"/>
        <Add filter="interface_polar_sasa"/>
        <Add filter="new_buns_sc_heavy"/>
       <Add filter="new_buns_bb_heavy" />
    </PROTOCOLS>
    <OUTPUT />
</ROSETTASCRIPTS>
```

In addition to 10-mer peptides, both smaller and larger scaffolds were also considered. However, 8- and 9-mer peptides showed in general reduced binding energies. For larger cycles, on the other hand, the entropic cost of folding to a single conformation is notably increased.[15]

The additional files that are needed for the script are

i) File L_res.txt, used to specify L-amino acids for design:

```
NOTAA G
start
```

ii) File D_res.txt, used to specify D-amino acids for design:

```
EMPTY NC DAL NC DAS NC DCS NC DPH NC DGU NC DHI NC DIL NC DLY NC DLE NC DME NC
DAN NC DGN NC DAR NC DSE NC DTH NC DVA NC DTR NC DTY NC DPR

start
```

iii) File limit_ALA.comp, to limit Ala residues in the peptide:

```
PENALTY_DEFINITION
TYPE ALA DAL

# Set how many residues you can be below the desired quantity before a penalty
is applied.
DELTA_START -1

# Set how many residues you can be above the desired quantity before a penalty
is applied.
DELTA_END 1

# Set the penalty for having too few, at the desired number, and too many of the
specified residues
PENALTIES 0 0 200

# Declare desired quantity of these residues
ABSOLUTE 1

#set how the penalties are applied
BEFORE_FUNCTION CONSTANT
AFTER_FUNCTION QUADRATIC
END_PENALTY_DEFINITION
```

iv) File bonus_PRO.comp, to favor Pro residues in the peptide:

```
PENALTY_DEFINITION
TYPE PRO DPR

# Set how many residues you can be below the desired quantity before a penalty
is applied.
DELTA_START -1

# Set how many residues you can be above the desired quantity before a penalty
is applied.
DELTA_END 1

# Set the penalty for having too few, at the desired number, and too many of the
specified residues
PENALTIES 200 0 0

# Declare desired quantity of these residues
ABSOLUTE 1

#set how the penalties are applied
BEFORE_FUNCTION QUADRATIC
AFTER_FUNCTION CONSTANT
END_PENALTY_DEFINITION
```

Energy landscape calculation of peptide macrocycles

Based on energy score and interaction metrics, the best results from the previous design step were subjected to independent energy landscape calculations using the simple_cycpep_predict application, as previously reported.[1]

General conditions for peptide synthesis

Peptides were synthesized using Fmoc/*t*Bu solid phase peptide synthesis (SPPS) using a CEM Liberty Blue microwave peptide synthesizer.

Peptides were purified by semi-preparative HPLC on a Waters 2700 sample manager equipped with a Waters 2487 dual-wavelength absorbance detector, a Waters 600 controller, a Waters fraction collector and Masslynx software by using a Sunfire C18 column (150 x 10 mm x 3.5 µm, 100 Å, Waters), flow rate 6.6 mL/min, solvent A=0.1% TFA in water; solvent B=0.1% TFA in acetonitrile.

Characterization was performed by UPLC (Waters Acquity equipped with Acquity photodiode array detector, flux rate 0.610 ml/min, Acquity UPLC BEH C18 Column, 130 Å, 1.7 µm, 2.1 mm x 100 mm), solvents A=0.045% TFA in water, and B=0.036% TFA in acetonitrile. Mass spectrometry was determined by UPLC-MS (Waters Acquity UPLC System equipped with ESI-SQ Detector2, flux rate 0.610 ml/min, Acquity UPLC BEH C18 Column, 130 Å, 1.7 µm, 2.1 mm x 100 mm), solvents A=0.1% formic acid in water, and B=0.07% formic acid in acetonitrile.

Synthesis of cyclic peptides **PD-*i*1-7**

Synthesis was performed on a Wang polystyrene resin (theoretical substitution of 1.14 mmol/g). Resin was swelled in 9:1 v/v CH_2Cl_2/DMF (15 mL/g). In a separate flask, Fmoc-Asp-OAll (1.2 eq, relative to the resin) and HOBt-Cl (1.2 eq) were dissolved in a minimum amount of DMF and added to the resin. Then, 4-dimethylaminopyridine (0.1 eq) and DIC (1.2 eq) were added. The mixture was allowed to react in an orbital shaker overnight at room temperature. Then, peptide chains were elongated by microwave-assisted automatic peptide synthesis. Fmoc deprotection

was carried out using 10% (w/v) piperazine and 0.1 M OxymaPure in a 9:1 mixture of NMP and EtOH. The N-Fmoc-protected amino acids (5 equiv, 0.2 M in DMF) were added with OxymaPure (5 equiv, 1 M in DMF) and DIC (5 equiv, 0.5 M in DMF) to the resin. The mixtures were stirred for 3 min at 90ºC, except for cysteines, histidines and arginines, which were coupled at 50ºC for 10 min. When the chain elongation was finished, Alloc groups were deprotected with phenyl silane (20 eq) and $Pd(PPh_3)_4$ (0.1 eq) in DCM (3 x 15 min treatments). On-resin peptide cyclization was performed with OxymaPure (5 eq) and DIC (5 eq) in DCM until the reaction was complete (typically, 16 h). Finally, peptides were cleaved with concomitant removal of side-chain protecting groups, using TFA, H_2O and TIS (92.5:5:2.5) for 2 h. After evaporating the residual TFA, peptides were ether precipitated and further purified by semi-preparative HPLC. Peptide characterization was performed by UPLC and UPLC-MS (see above).

Recombinant expression of human PD-1

Procedure adapted from ref.[2] Genes encoding the interacting ectodomain of human PD-1 (aa 33-150) was cloned into pET-24d. A Cys to Ser mutation was introduced at position 93 of PD-1 to aid protein stability and folding. The protein was expressed in *E. coli* BL21(DE3) in the form of inclusion bodies. The cells were grown at 37 °C in LB medium supplemented with 50 µg/mL kanamycin until OD_{600} reached 0.6–1.0, and the protein expression was induced with 1 mM IPTG and incubated for 16 h at 30 °C. The cells were harvested by centrifugation, re-suspended in PBS (containing protease inhibitor cocktail and DNAse) and lysed by sonication on ice. Inclusion bodies were recovered by centrifugation (25,000 g for 30 min at 4 °C) and washed 2 times with 50 mM Tris buffer (200 mM NaCl, 0.5% Triton X-100, 10 mM EDTA and 10 mM 2-mercaptoethanol, pH 8.0) followed by a final wash with the same buffer without the detergent. The inclusion bodies were re-suspended in guanidinium hydrochloride buffer (50 mM Tris, 6 M GuHCl, 200 mM NaCl, 10 mM 2-mercaptoethanol, pH 8.0) by stirring vigorously for 2 h at 4ºC. After removing undissolved residue by centrifugation (25,000g for 30 min at 4 °C) and refolded by slow drop-wise dilution in folding buffer (0.1 M Tris, 0.4 M L-Arg hydrochloride, 2 mM EDTA, 5 mM cystamine, 0.5 mM cysteamine, pH 8.0) for 3 days at 4ºC. The protein was then dialyzed 2 times against 10 mM Tris pH, 20 mM NaCl, pH 8.0 buffer. The solubilized fraction was diluted 1:2 in water, acidified to pH 6 and applied to HiTrap SP HP cation exchange chromatography column (GE Healthcare Life Sciences). The protein was washed with five column volumes of wash buffer (30 mM NaCl, 0.7 mM KCl, 2.5 mM phosphate buffer, pH 6.0) and eluted using a 0-50% gradient of elution buffer (1 M NaCl, 0.7 mM KCl, 2.5 mM phosphate buffer, pH 6.0). Finally, the protein was purified by gel filtration chromatography using a HiLoad 16/60 Superdex 200 pg column (GE Healthcare Life Sciences) with 10 mM Tris, 20 mM NaCl, pH 8.0 buffer. The protein purity was evaluated by SDS-PAGE, high-resolution mass spectrometry and multiangle light scattering.

^{15}N-labeled hPD-1 was produced likewise from *E. coli* cells grown in M9 minimal medium containing $^{15}NH_4Cl$ as the sole nitrogen source.

Surface plasmon resonance (SPR)

Binding experiments were carried out in a Biacore T200 system (GE Healthcare) at 25ºC in PBS-T buffer (0.05% Tween 20, pH 7.4). hPD-1 (10 µg/mL) at 25 µg/ml in 10 mM sodium acetate (pH 5.5) was directly immobilized on the dextran matrix of a CM5 sensor chip (GE Healthcare) by amine coupling using the manufacturer's kit (GE Healthcare) and an injection time of 80 sec, resulting in immobilization levels of ~600 RU. A solution of 10 mM glycine-HCl at pH 2.5 was used for chip regeneration between analyte injections. The protein bioactivity was assessed by injecting serial dilutions of Fc-tagged PD-L1 (Peprotech #310-35) at 1-min injections and performing kinetic analysis of the sensorgrams, which yielded a K_D value of 4.6 µM for the interaction, in agreement with previous results.[3] To assess the potential interaction of peptides **PD-*i*1-7**, analytes were dissolved in PBS and injected at 10, 100 and 1000 µM concentrations. K_D values could not be unequivocally calculated due to their fast kinetics and lack of signal saturation. Due to this limitation, those peptides presenting clear concentration-dependent association-dissociation signals were further evaluated by MST.

Microscale thermophoresis (MST)

MST was used to calculate the binding affinities in solution of **PD-*i*3** and **PD-*i*6** for the extracellular domain of human PD-1. To this end, PD-1 was fluorescently labelled by reaction of the Lys side chains with Alexa-647-NHS (6 eq) for 3 h at 4ºC. The labelled protein was purified by size-exclusion chromatography and was diluted in PBS-T buffer (0.05% Tween 20, pH 7.4) to a final concentration of 10 nM. MST binding assays were performed on a Monolith NT.115 instrument (NanoTemper Technologies) with standard capillaries at 25ºC. The protein bioactivity was confirmed by using Fc-PD-L1 (Peprotech #310-35) and a specific anti-PD1 mAb (BioCell #BE0193) as positive controls (Figure S). Peptides were diluted 2:1 from a concentrated stock in 10 µL of PBS-T buffer to make a 16-sample dilution series, which were titrated against a constant concentration of PD-1 (10 nM). A reproducible and negative thermophoresis response was observed, which was baseline-corrected and normalized (ΔFnorm [‰]).The curves were analyzed using a standard Langmuir binding model, from which dissociations constants (K_D) were determined. The experiment was performed in triplicate.

Isothermal titration calorimetry (ITC)

ITC experiments were performed at 25ºC using a low-volume nano ITC calorimeter (TA instruments). Ligand and protein samples were dissolved in the same buffer (10 mM Tris, 20 mM NaCl, pH 8.0), centrifuged, and degassed prior to the ITC experiments. For each titration, a concentrated peptide solution was injected into a cell containing 190 µL of protein solution at a concentration of 30 µM. A total of 16 injections of 3 µL per titration were performed with a 4-min delay after each injection. Binding isotherms were analysed using the software provided by TA instruments, assuming a single binding site for the independent domains. Baseline controls were acquired with buffer and pure peptide solutions.

NMR spectroscopy

^{1}H,^{15}N HSQC spectra were recorded at 25ºC on a Bruker 600 MHz spectrometer equipped with a cryoprobe. ^{15}N-labelled PD-1 (60 µM) was prepared in NMR buffer (25 mM potassium phosphate, 100 mM NaCl, 10% D_2O, pH 6.4). Spectra were acquired with 180x2048 complex points with a total of 48 transients per increment. ^{1}H chemical shifts were referenced to internal DSS (4,4-dimethyl-4-silapentane-1-sulfonic acid). Data processing was performed using TopSpin v3.0. Assignment of the human PD-1 ^{1}H,^{15}N HSQC spectrum has been previously reported.[3] Relative signal intensity changes (I/Io) due to the addition of macrocyclic peptides (1 mM) were plotted for each protein residue, I/Io > 20% were considered significant.

To assess the solution structures of **PD-*i*3** and **PD-*i*6**, NMR spectra were recorded at 5ºC on a Bruker B800 MHz spectrometer equipped with a cryoprobe. The NMR sample was prepared by dissolving the peptide in NMR buffer at a final concentration of 1 mM. ^{1}H chemical shifts were referenced to internal DSS. Data processing was performed using TopSpin v3.0. Complete proton and carbon resonance assignment was obtained by the combined analysis of 2D homo- (TOCSY, NOESY) and natural abundance hetero-nuclear ^{1}H-^{13}C HSQC experiments. The TOCSY and NOESY mixing times were 70 and 200 ms, respectively. Suppression of the water signal was achieved by excitation sculpting. C_α and H_α secondary chemical shifts (Δδ) were calculated as the difference between the measured chemical shift ($\delta_{measured}$) and reported values for random coil (δ_{RC}).[4]

NMR structural determination

To calculate the structure of **PD-*i*3** and **PD-*i*6** in solution, simulated annealing calculations were performed with the Xplor-NIH software,[5] which can handle L- and D-isomers and cyclic backbones. Distance restraints derived from NOEs observed in the ^{1}H,^{1}H-NOESY spectrum were sorted into strong (2.5 0.7 2.0), medium (3.0 1.2 2.0) and weak (4.0 2.2 2.0), according to their relative intensities. A square-well potential was used to model these restraints. Amide proton temperature coefficients were calculated to infer the presence of backbone hydrogen bonds.[6] Dihedral angles

were left unrestrained due to the lack of sufficient information for handling D-amino acids. A total of 1000 structures were calculated and sorted by total energy. The refined coordinates for the ensemble of 10 lowest-energy conformations are available at the Protein Data Bank under accession codes 6TVJ (for **PD-*i*3**) and 6TT6 (for **PD-*i*6**).

Molecular dynamics (MD)

Unrestrained MD simulations were performed with the Amber14 software using the ff14SB forcefield. The lowest energy structure of the NMR-calculated ensemble was solvated using the Leap module in a pre-equilibrated octahedral box of TIP3P water molecules. Chlorine or sodium ions were added to obtain an electrostatically neutral system. The initial complex structure was first subjected to a minimization protocol consisting of 1000 steps of steepest decent method followed by 500 steps of conjugate gradient method. Thermalization of the system was performed in the NVT ensemble during 200 ps, using a time step of 1 fs and increasing the temperature from 100 to 298 K, where a force constant of 5 kcal mol^{-1} $Å^{-2}$ was applied to protein backbone atoms. Prior to the production run, a short MD simulation (100 ps) in the NPT ensemble was done in order to equilibrate the system density to 1 atm and 298 K. From each equilibrated system, 3 simulations of 100 ns were performed at constant pressure (1 atm) and temperature (298 K) using periodic boundary conditions. Low harmonic constraints (2 kcal mol^{-1} $Å^{-2}$) were used to reduce the protein mobility. Constant temperature was achieved using the Langevin thermostat with a collision frequency of 2 ps^{-1}. The SHAKE algorithm was used to keep bonds involving hydrogen atoms at their equilibrium length. The particle-mesh Ewald summation method was used to deal with long range electrostatic interactions and a cut-off of 10 Å was applied for non-bonded interactions. Frames collected every 2 ps were analyzed using the CPPtraj module of Amber. The root-mean-square deviation (RMSD) of the position of the Cα of the peptides was calculated for each frame and compared to the target design.

Supporting figures

Figure S1

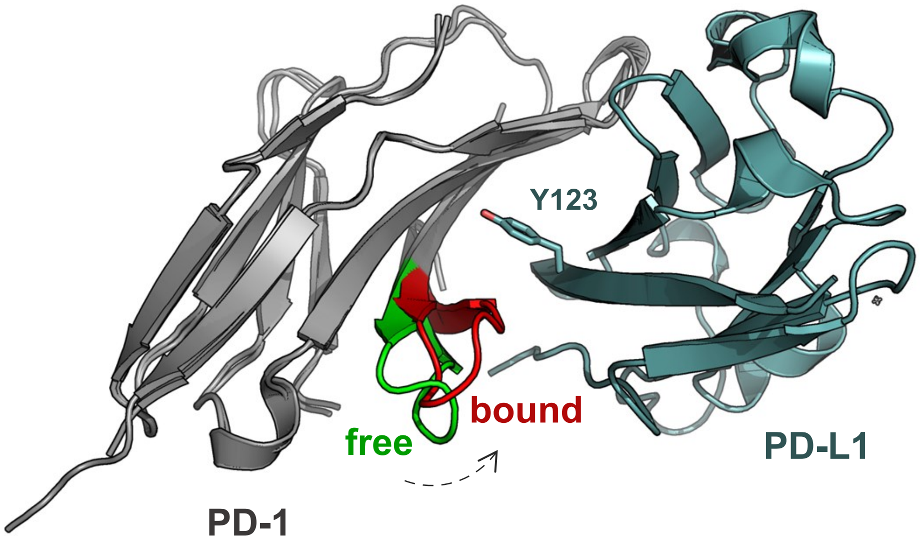

Figure S1. Overlay of ribbon diagrams of the apo (PDB ID: 3RRQ) and PD-L1-bound (PDB ID: 4ZQK) forms of human PD-1. Binding of PD-L1 induces a rearrangement of the β-sheet interface of PD-1. The CC' loop (residues M70-D77) is highlighted for the apo (green) and PD-L1-bound (red) structures. The Y123 hotspot residue is shown as sticks.

Figure S2

A)

```
Q9NZQ7 PD1L1_HUMAN    117   SYGGA-DYKRITVKVNAPYNKINQRILVVDPVTSEHELTCQAEGYPKAEVIWTSSDHQVL
Q9BQ51 PD1L2_HUMAN    105   IYGVAWDYKYLTLKVKASYRKINTHILK-VPETDEVELTCQATGYPLAEVSWPNVS----
Q9EP73 PD1L1_MOUSE    117   SYGGA-DYKRITLKVNAPYRKINQRI-SVDPATSEHELICQAEGYPEAEVIWTNSDHQPV
Q9WUL5 PD1L2_MOUSE    105   ICGAAWDYKYLTVKVKASYMRIDTRILE-VPGTGEVQLTCQARGYPLAEVSWQNVS----
D4AE25 D4AE25_RAT     117   SYGGA-DYKRITLKVNAPYRKINQRI-SMDPATSEHELMCQAEGYPEAEVIWTNSDHQSL
D4AAV6 D4AAV6_RAT     105   ICGAAWDYKYLTVKVKASYVRIDTGILE-VPGTGEVQLICQARGYPLAEVSWQNVS----
E2RKZ5 E2RKZ5_CANLF   117   GYGGA-DYKRITLKVHAPYRNISQRI-SVDPVTSEHELMCQAEGYPEAEVIWTSSDHRVL
F1PSG6 F1PSG6_CANLF   105   LYGVAWDYKYLTLKVKASYKKINTHFLR-IPGLDEVEFTCQAEGYPLAEVFWPNVS----
```

B)

pdb#	chain	int_id	aa	DDG(complex)
19	A	0	5	-0.05
23	A	1	18	0.15
26	A	1	3	0.92
54	A	1	8	0.93
56	A	1	20	4.05
58	A	1	4	2.34
63	A	1	12	1.06
66	A	1	14	2.26
68	A	0	18	0.21
76	A	0	18	0.27
113	A	1	15	1.76
115	A	1	11	1.13
117	A	1	16	-0.02
122	A	1	3	0.88
123	A	1	20	3.77
124	A	1	9	1.46
125	A	1	15	0.31

C)

Residue		Score
A19	F	-0.278
A20	T	-0.638
A23	V	-0.928
A26	D	-0.929
A54	I	-1.121
A56	Y	-0.323
A58	E	-0.778
A63	N	-1.357
A66	Q	-0.786
A76	V	-1.448
A113	R	1.678
A115	M	-0.183
A117	S	-1.169
A122	D	0.755
A123	Y	1.638
A124	K	0.567
A125	R	-0.736
A126	I	-1.030

Figure S2. A) Amino acid sequence alignment of PD-1 ligands across different species, showing conservation of the hotspot Tyr residue. B and C) Predicted changes in binding free energy (ddG) upon alanine mutation, for PD-L1 residues, computed using the Robetta server[7] and the HSPred algorithm.[8]

Figure S3

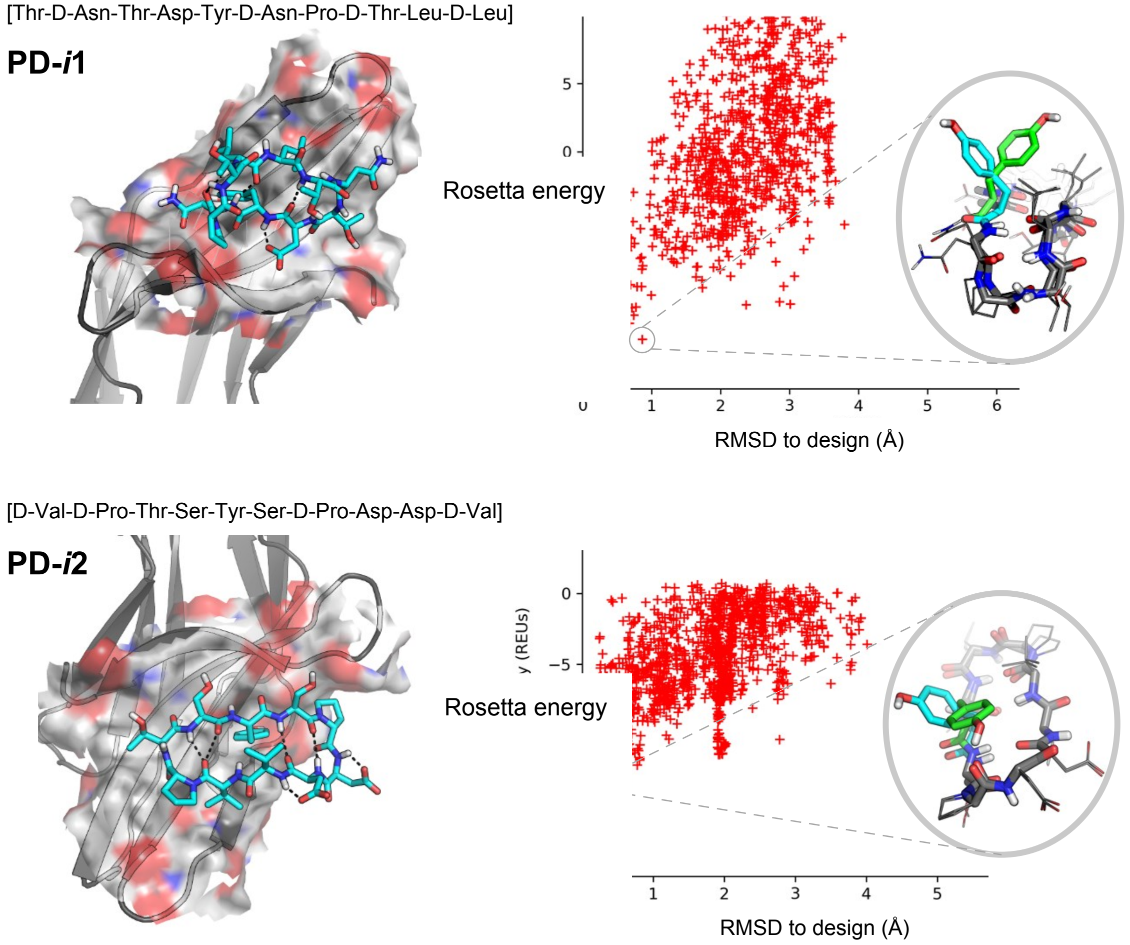

Figure S3. Structures and sequences of designed peptides **PD-*i*1** and **PD-*i*2** in complex with PD-1 (left). Energy landscape calculations (right) show convergence towards low-energy structures that are close to the design (backbone RMSD < 1 Å); preferred rotamers for the Tyr side chain are however very different (see inset, lowest-energy energy structure in green, target design in blue).

Figure S4

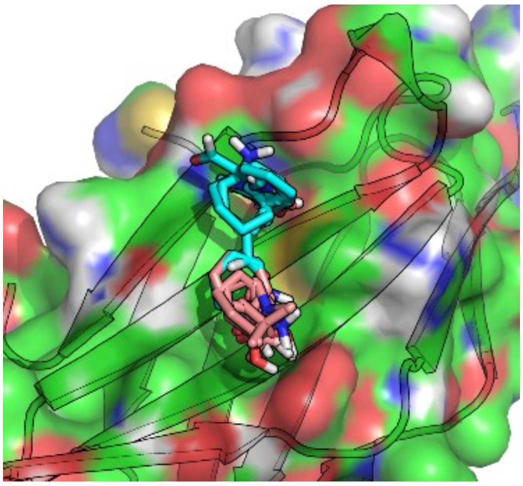

Figure S4. Mapping of binding regions in apo-PD-1 (PDB ID: 3RRQ) using the FTMap server.[9] FTMap samples positions of small molecules as probes and scores and clusters the poses using a detailed energy expression. The main cluster #1 (in cyan) and cluster #4 (in pink) are both located on the PD-L1 binding interface and were used to anchor hotspot residues for designing peptides **PD-*i*5-7**.

Figure S5

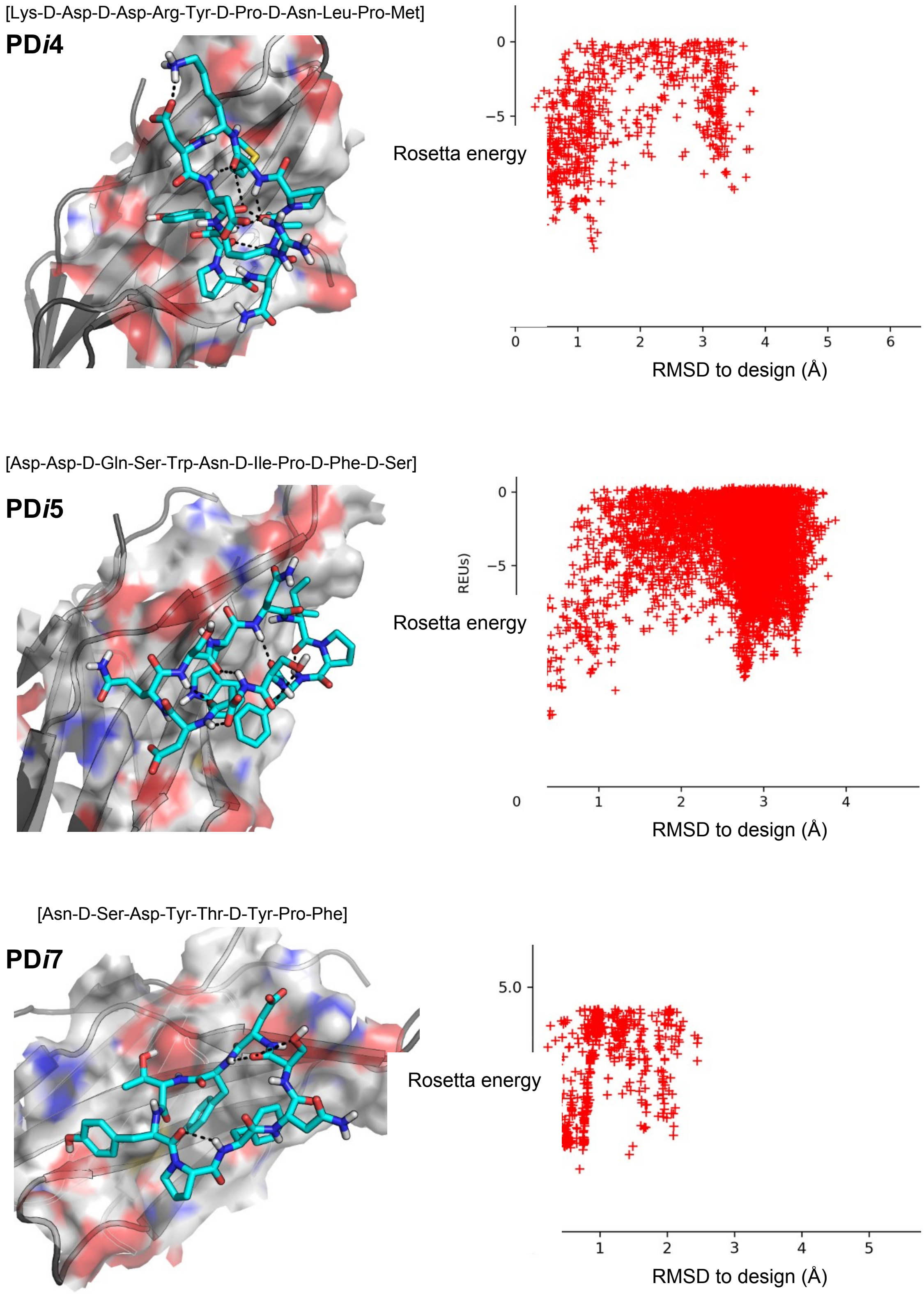

Figure S5. Structures and sequences of designed peptides **PD-*i*4**, **PD-*i*5** and **PD-*i*7** in complex with PD-1 (left). Energy landscape calculations (right) show convergence towards low-energy structures that are close to the design.

Figure S6

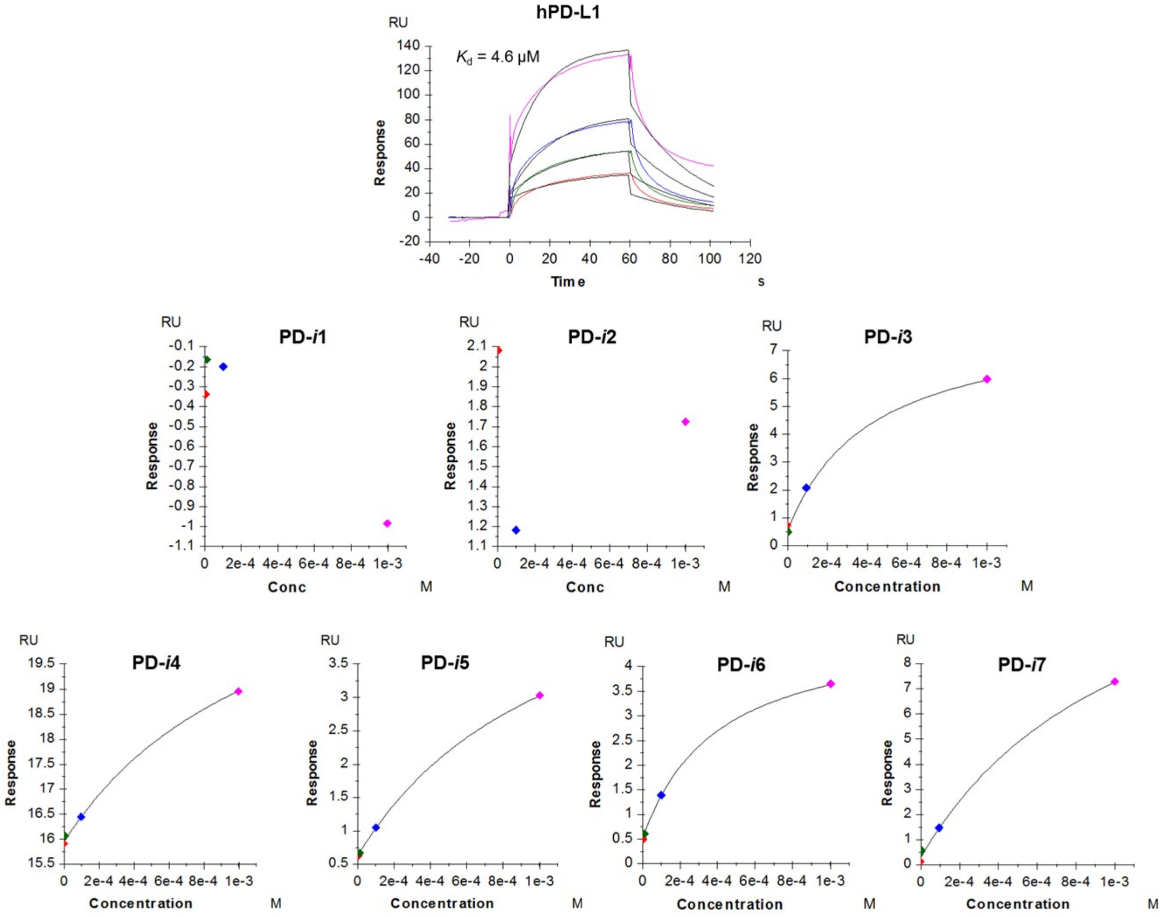

Figure S6. SPR curves for human PD-L1 (control) and **PD-*i*1-7** injected as analytes at 25ºC through a flow cell containing immobilized PD-1 (background responses from reference channel subtracted). Kinetic analysis of PD-L1 titration yielded a K_D value in agreement with previous results.[3] Affinity analysis was performed for **PD-*i*1-7**.

Figure S7

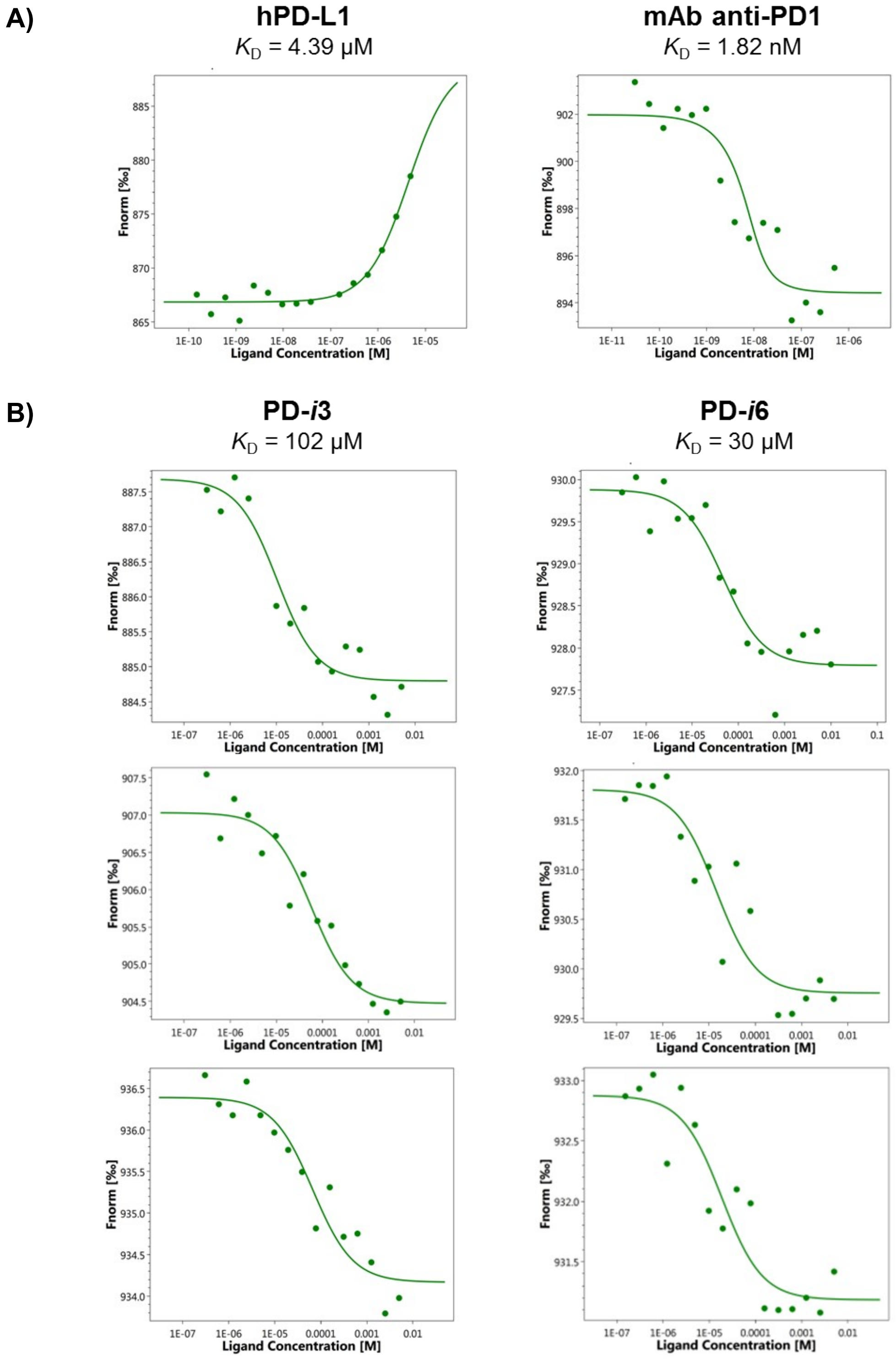

Figure S7. Normalized MST signals for the interaction of PD-1 with (A) human PD-L1 and an anti-PD1 mAb (as controls) and with (B) macrocyclic peptides **PD-*i*3** and **PD-*i*6**. Experimental triplicates are shown.

Figure S8

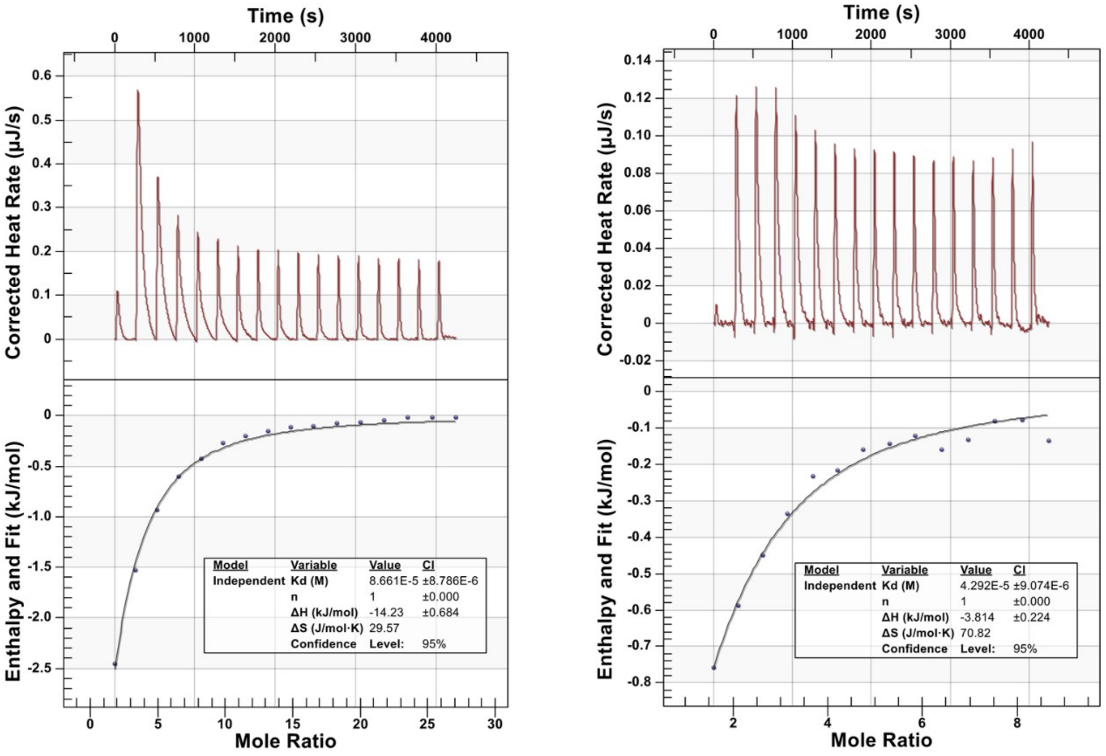

Figure S8. Isothermal titration calorimetry (ITC) curves of PD-1 titrated with **PD-*i*3** (left) and **PD-*i*6** (right). Data points were fitted to a 1:1 Langmuir binding model.

Figure S9

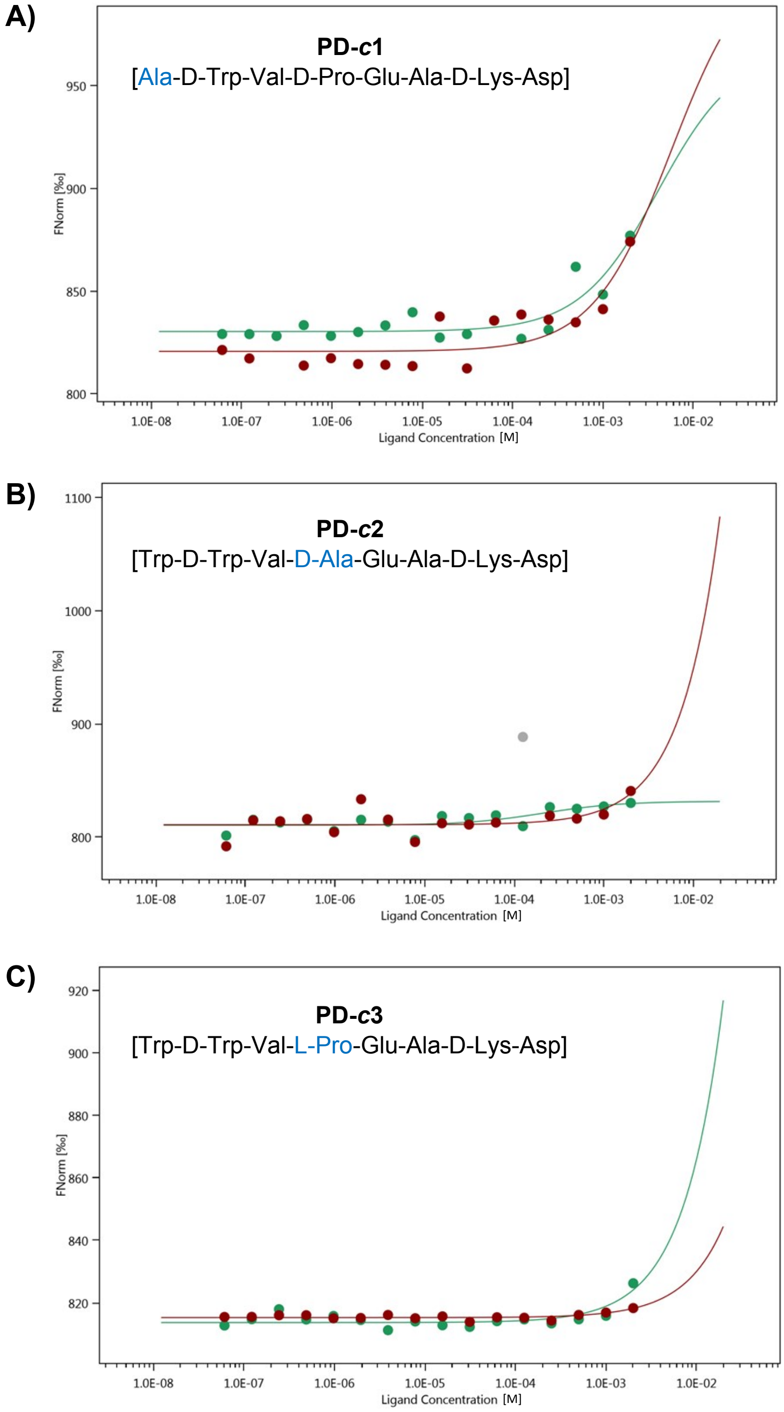

Figure S9. Normalized MST signals for the interaction of PD-1 with three mutated negative control versions of **PD-*i*6** (the mutated residue is highlighted in blue). A) **PD-*c*1**, in which the Trp anchor residue is mutated to Ala; B) **PD-*c*2**, in which D-Pro is mutated to D-Ala; and C) **PD-*c*3**, in which D-Pro is mutated to L-Pro. Experimental duplicates are shown (fitting curves are only indicative).

Figure S10

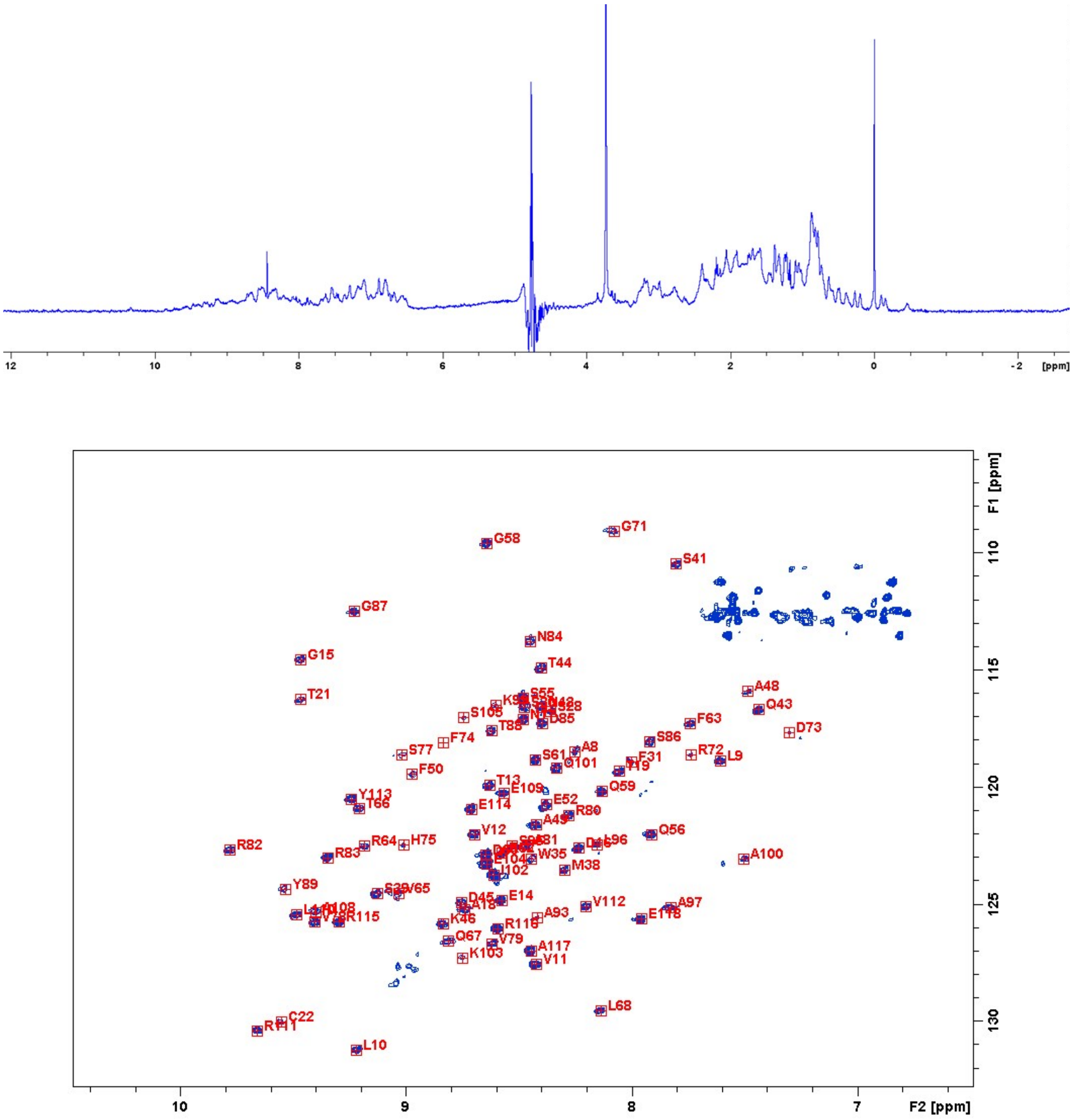

Figure S10. ^{1}H and $^{1}H,^{15}N$ HSQC spectra of ^{15}N-labelled PD1 (60 µM) recorded at 25ºC in 25 mM potassium phosphate, 100 mM NaCl, 10% D_2O buffer, pH 6.4. Assigned residues are shown.

Figure S11

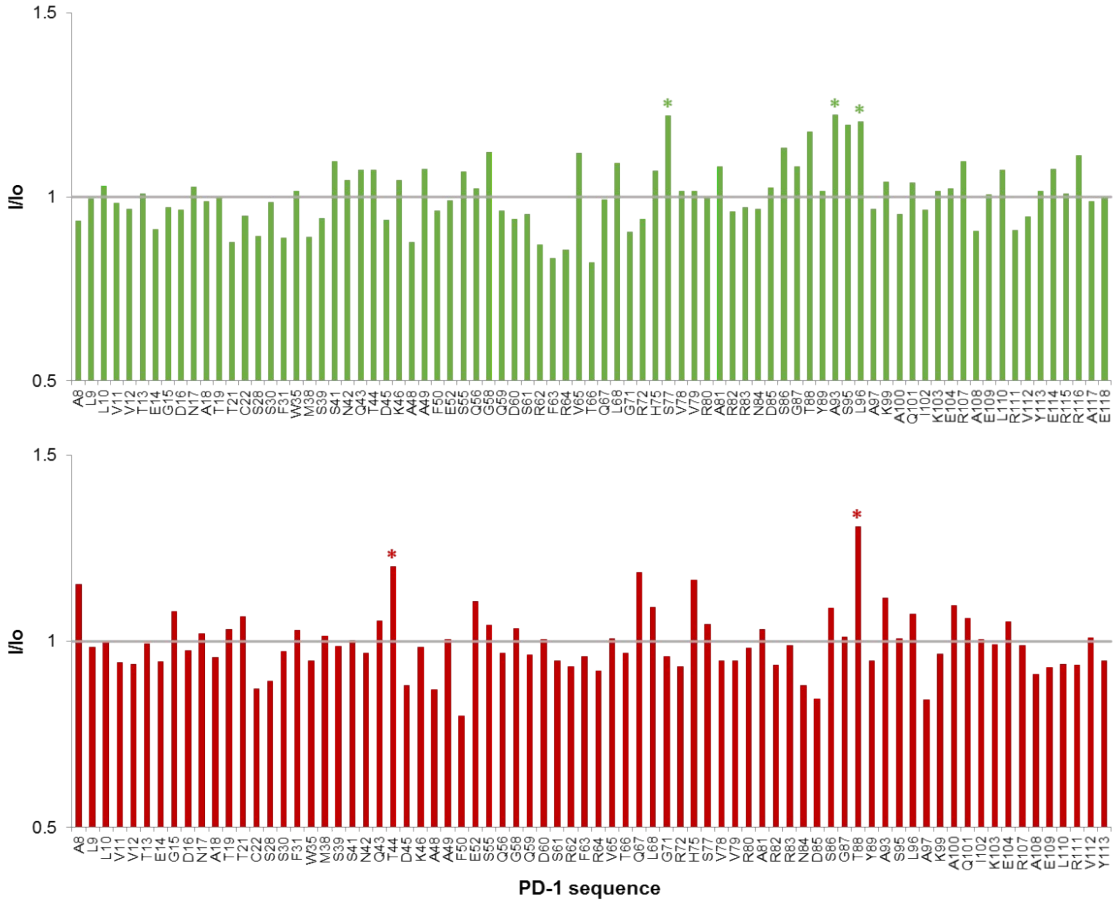

Figure S11. Relative intensity changes in the ^{1}H,^{15}N HSQC spectra of ^{15}N-labelled PD1 (60 μM) plotted for each protein residue caused by the addition of **PD-*i*3** (top) and **PD-*i*6** (bottom). Residues highlighted (*) are those represented in sticks in Figure 2B.

Figure S12

A)

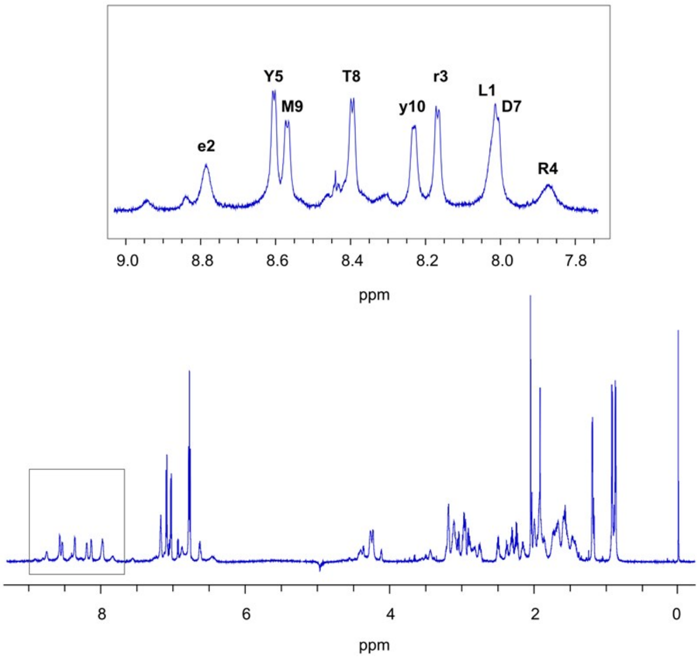

B)

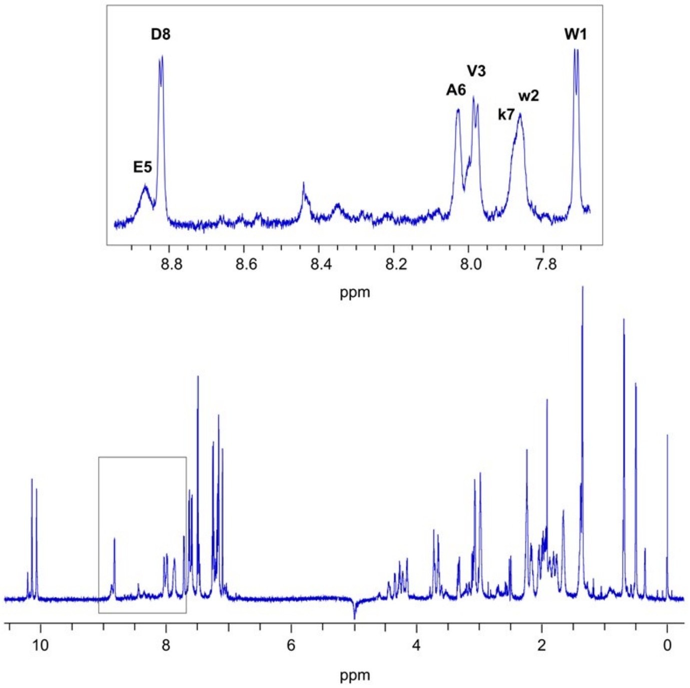

Figure S12. ^{1}H-NMR spectra of **PD-*i*3** (A) and **PD-*i*6** (B) showing a single set of ^{1}HN backbone signals.

Supporting data

UPLC-MS of peptides **PD-*i*1-7**

UPLC chromatography was performed on a Waters Acquity system equipped with Acquity photodiode array detector, flux rate 0.610 ml/min, Acquity UPLC BEH C18 Column, 130 Å, 1.7 µm, 2.1 mm x 100 mm, solvents A=0.045 % TFA in water, and B=0.036 % TFA in acetonitrile (ACN). Mass spectrometry was determined by UPLC-MS on a Waters Acquity UPLC System equipped with ESI-SQ Detector2, flux rate 0.610 ml/min, Acquity UPLC BEH C18 Column, 130 Å, 1.7 µm, 2.1 mm x 100 mm), solvents A=0.1 % formic acid in water, and B=0.07 % formic acid in ACN.

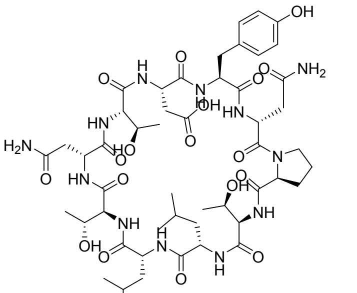

PD-*i*1

Thr-D-Asn-Thr-Asp-Tyr-D-Asn-Pro-D-Thr-Leu-D-Leu

Molecular Weight: 1133,22

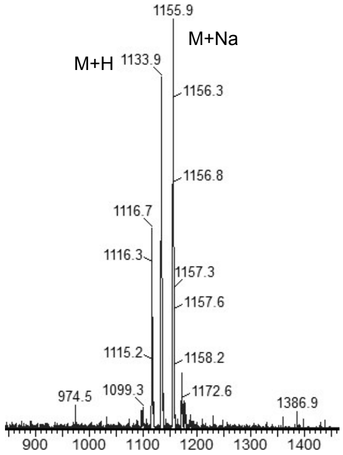

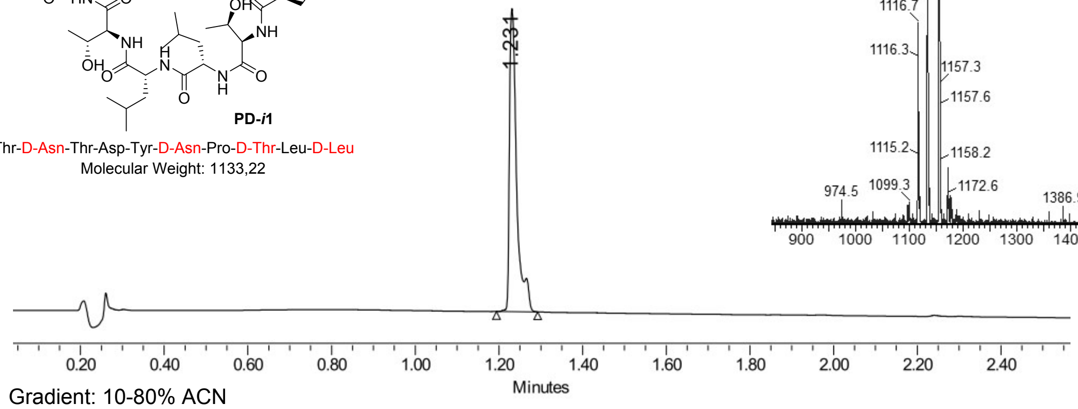

Gradient: 10-80% ACN

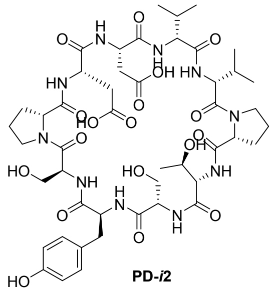

PD-*i*2

Molecular Weight: 1061,11

D-Val-D-Val-D-Pro-Thr-Ser-Tyr-Ser-D-Pro-Asp-Asp

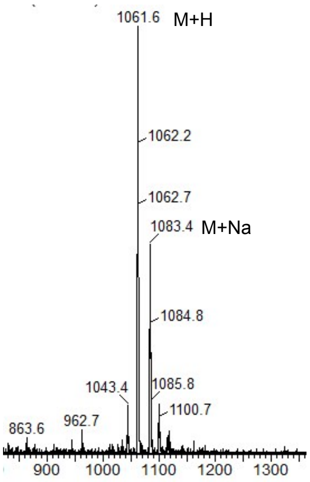

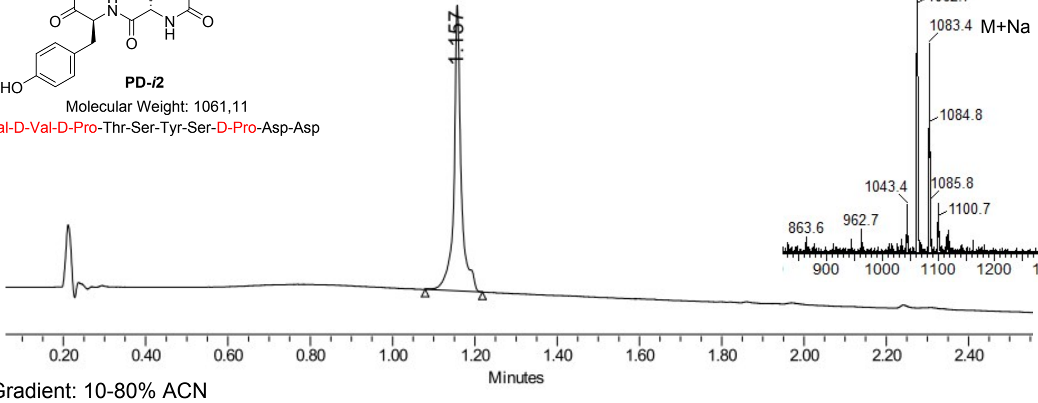

Gradient: 10-80% ACN

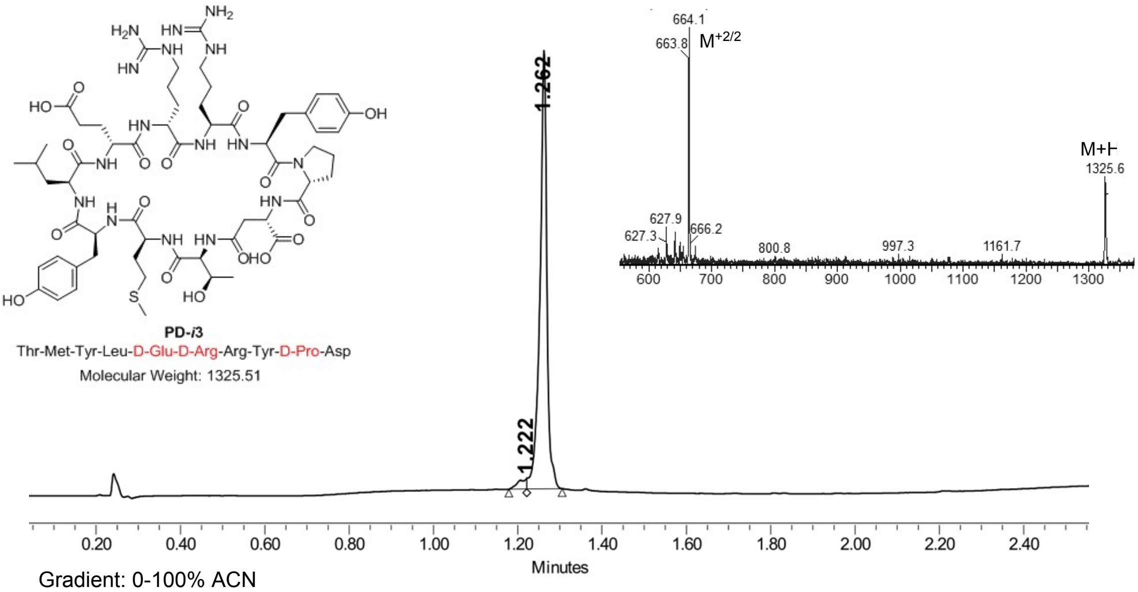

Gradient: 0-100% ACN

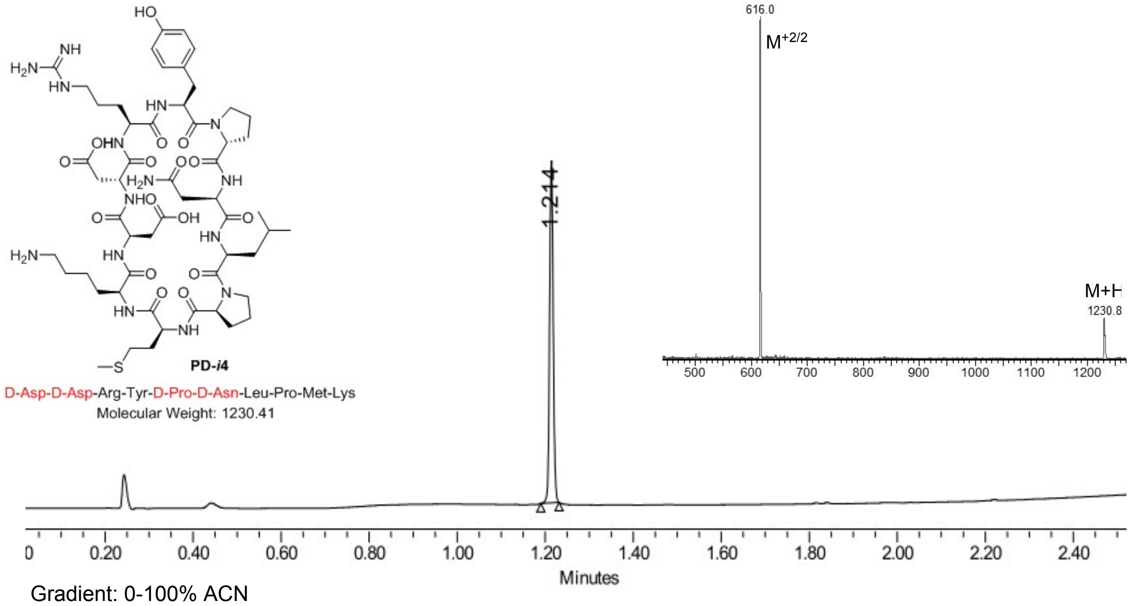

Gradient: 0-100% ACN

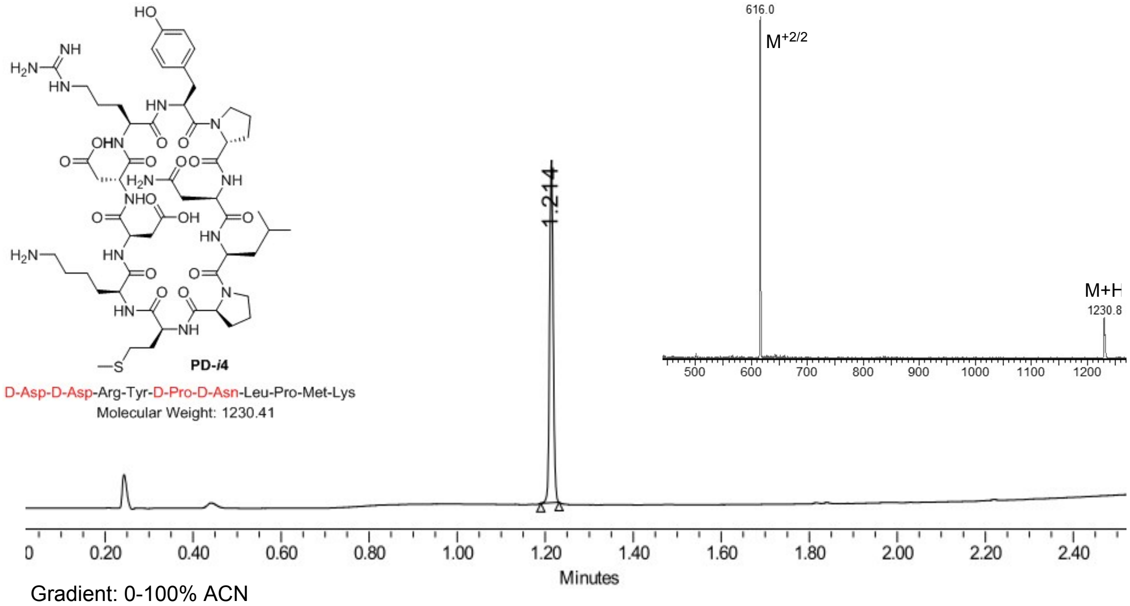

Gradient: 0-100% ACN

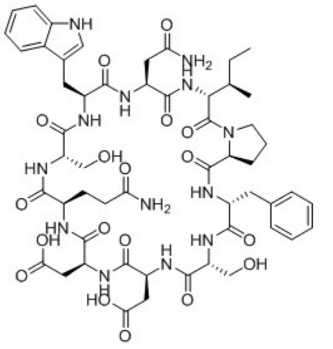

PD-*i*5
D-Gln-Ser-Trp-Asn-D-Ile-Pro-D-Phe-D-Ser-Asp-Asp
Molecular Weight: 1190.24

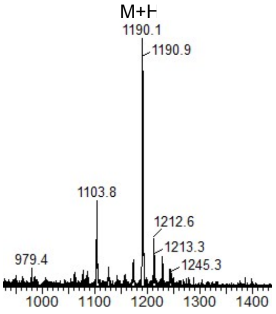

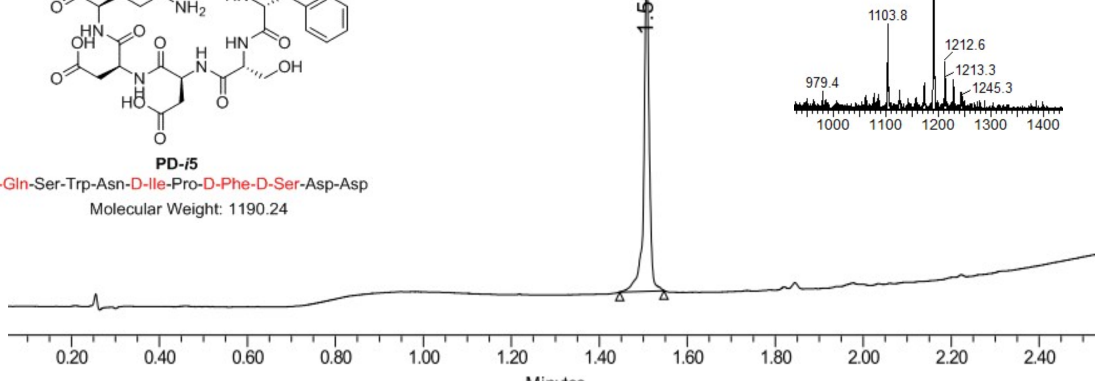

Gradient: 0-100% ACN

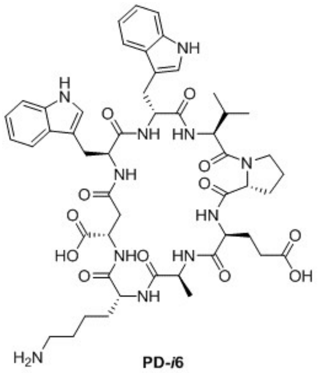

PD-*i*6
Trp-D-Trp-Val-D-Pro-Glu-Ala-D-Lys-Asp
Molecular Weight: 1012.14

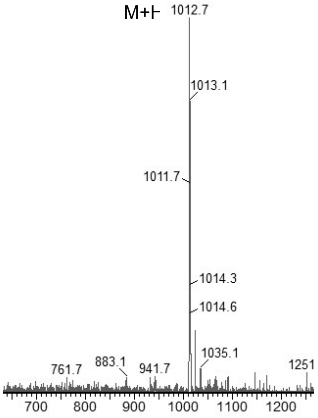

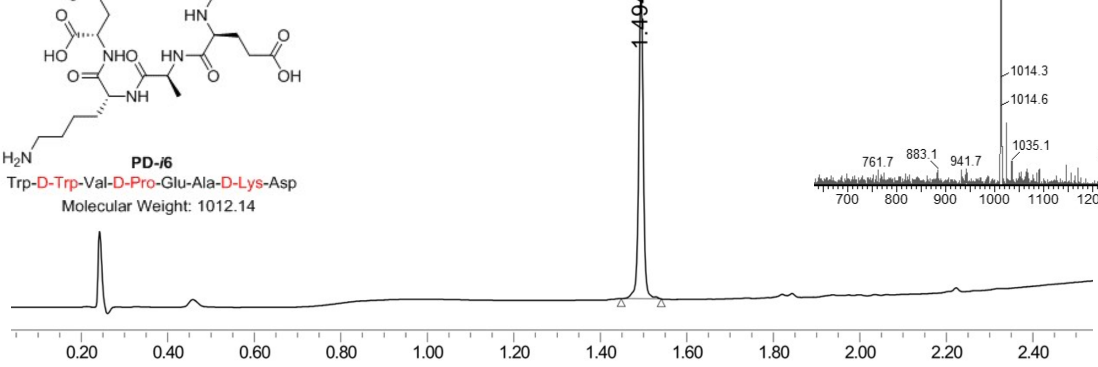

Gradient: 0-100% ACN

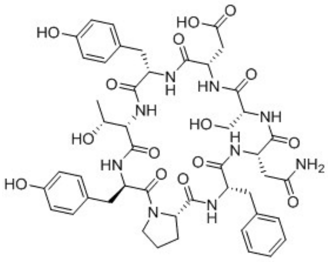

PD-*i*7
Tyr-Thr-D-Tyr-Pro-Phe-Asn-D-Ser-Asp
Molecular Weight: 988.02

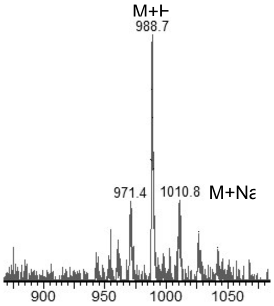

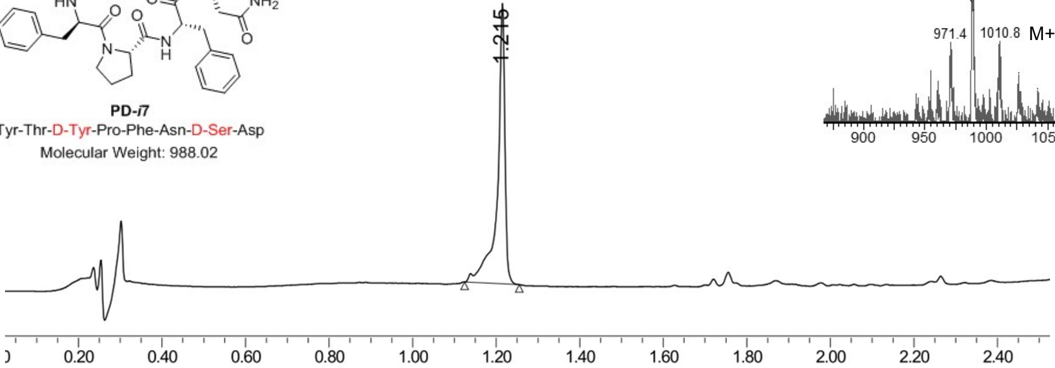

Gradient: 15-80% ACN

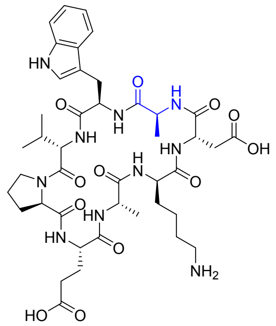

PD-*c*1
Ala-D-Trp-Val-D-Pro-Glu-Ala-D-Lys-Asp
Molecular Weight: 897.00

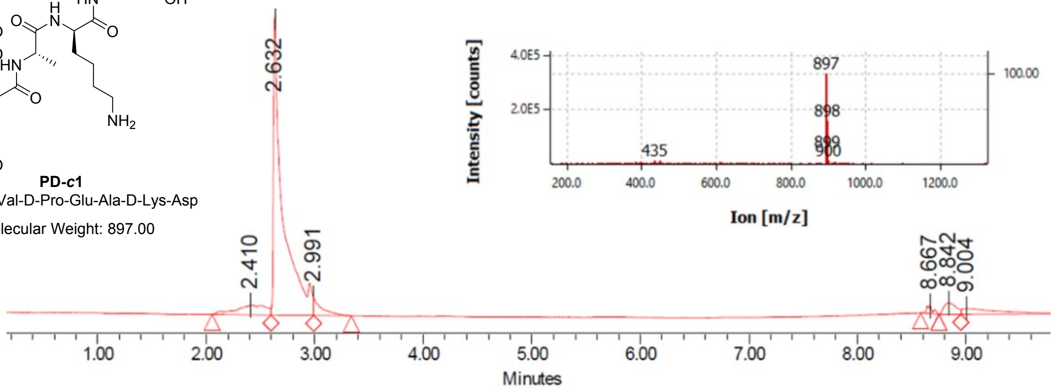

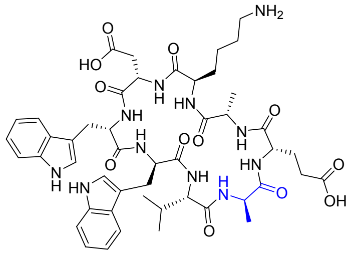

PD-*c*2
Trp-D-Trp-Val-D-Ala-Glu-Ala-D-Lys-Asp

Molecular Weight: 986.10

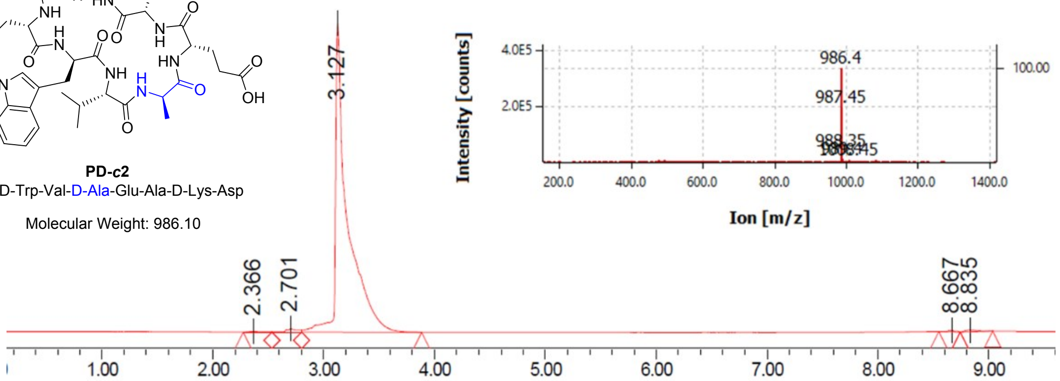

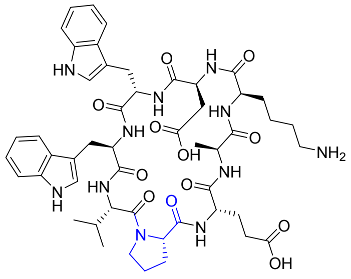

PD-*c*3
Trp-D-Trp-Val-L-Pro-Glu-Ala-D-Lys-Asp
Molecular Weight: 1012.14

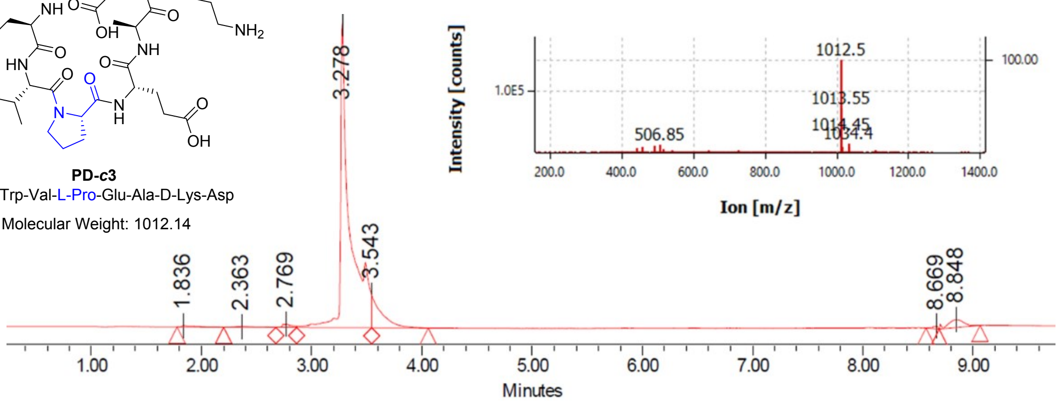

SEC-MALS and MS of recombinantly expressed human PD-1

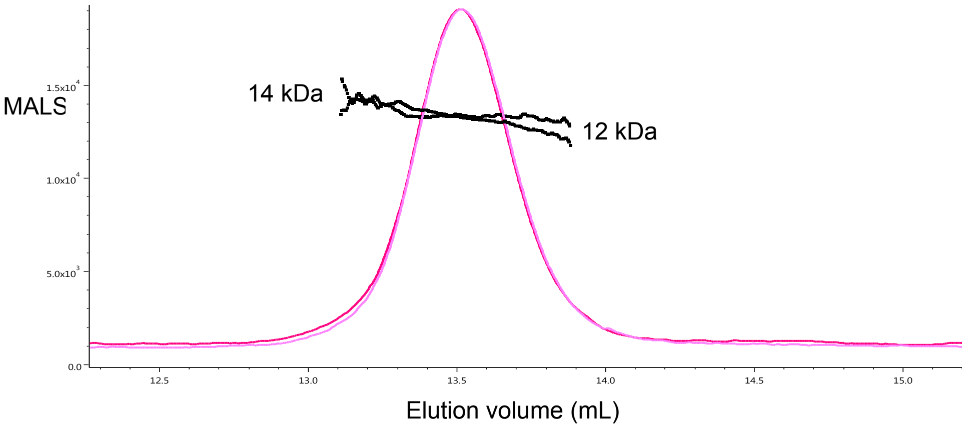

	Mn (kDa)	Mw (kDa)	Polydispersity
**PD-1**	13.39 ± 0.10	13.39 ± 0.10	1.00 ± 0.01
**BSA_standard**	67.66 ± 0.21	67.67 ± 0.21	1.00 ± 0.00

PD-1 sequence (extracelular domain):

```
         10         20         30         40         50         60
MPPTFSPALL VVTEGDNATF TCSFSNTSES FVLNWYRMSP SNQTDKLAAF PEDRSQPGQD

         70         80         90        100        110
SRFRVTQLPN GRDFHMSVVR ARRNDSGTYL CGAISLAPKA QIKESLRAEL RVTERRAE
```

Theoretical MW: 13227.83

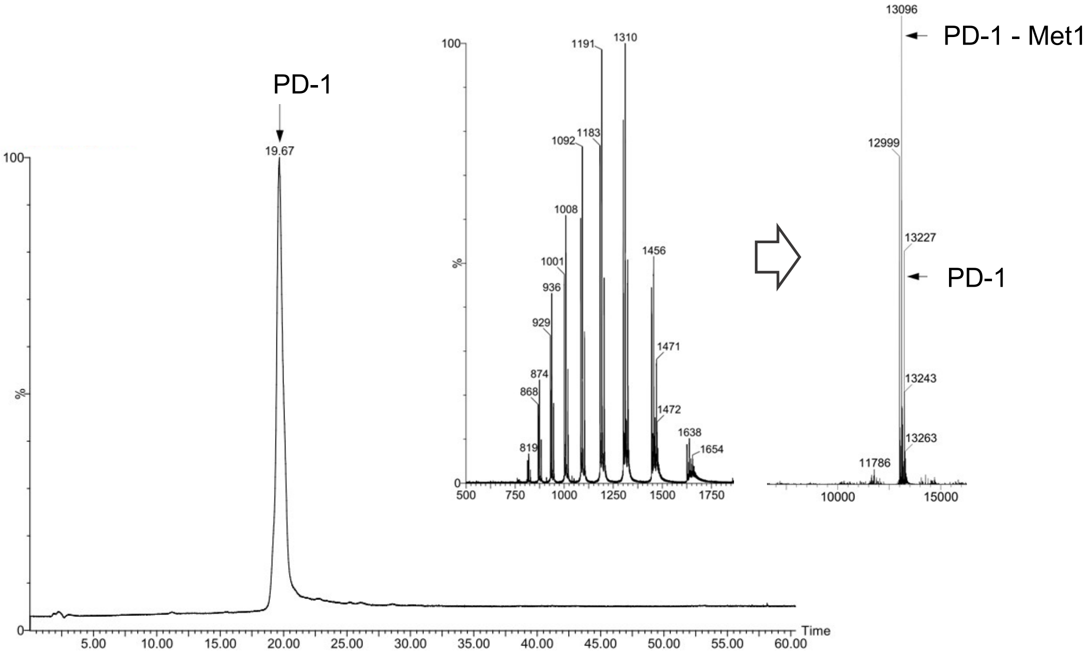

NMR assignment of **PD-*i*3** and **PD-*i*6**

PD-*i*3 (LerRYpDTMY)

Residue	Hα	Hβ	Cα	Cβ	NH	Others	
**Leu**	4,26	1,57	55,62	41,83	8,02	Hγ= 1,42<br>Hδ= 0,89-0,88	Cγ= 26,78<br>Cδ= 24,65-23,71
**D-Glu**	4,25	2,23-2,30	56,91	36,18	8,78	Hγ= 2,15-1,92	Cγ= 29,96
**D-Arg**	4,26	1,90-1,86	56,90-	30,69	8,16	Hγ= 1,66-1,56<br>Hδ= 3,17	Cγ= 27,30<br>Cδ= 43,31
**Arg**	4,38	1,73-1,70	55,73	31,46	7,86	Hγ= 1,53-1,46<br>Hδ= 3,11	Cγ= 27,11<br>Cδ= 43,27
**Tyr**	4,63	2,95-2,90	56,42	38,69	8,60	$H_{2,6}$ = 7,12<br>$H_{3,5}$ = 6,81	
**D-Pro**	4,36	1,92	63,38	31,94	-	Hγ = 1,72-1,65<br>Hδ= 3,43-2,81	Cγ= 26,20<br>Cδ= 50,04
**Asp**	4,40	2,95-2,74	55,25	40,77	8,00		
**Thr**	4,23	4,22	62,35	69,64	8,39	Hγ = 1,19	Cγ= 21,66
**Met**	4,42	1,99	55,95	32,16	8,56	Hγ = 2,49-2,37<br>HƐ = 2,04	Cγ= 32,03<br>CƐ= 16,69
**Tyr**	4,55	3,04-2,97	58,17	38,25	8,22	$H_{2,6}$ = 7,06<br>$H_{3,5}$ = 6,80	

PD-*i*6 (WwVpEAkD)

Residue	Hα	Hβ	Cα	Cβ	NH	Others	
**Trp**	4,58	3,06	57,93	30,64	7,71	H_1= 10,06<br>H_2= 7,15<br>H_4= 7,62<br>H_5= 7,18<br>H_6= 7,24<br>H_7= 7,48	 C_2= 122,21<br>C_4= 121,06<br>C_5= 121,94<br>C_6= 124,66<br>C_7= 114,73
**D-Trp**	4,60	3,32–3,09	56,89	28,97	7,85	H_1= 10,13<br>H_2= 7,09<br>H_4= 7,58<br>H_5= 7,15<br>H_6= 7,24<br>H_7= 7,48	 C_2= 127,38<br>C_4= 120,90<br>C_5= 121,91<br>C_6= 124,66<br>C_7= 114,69
**Val**	4,48	1,93	59,22	32,18	7,97	Hγ= 0,49-0,68	Cγ= 19,12-21,08
**D-Pro**	4,34	2,25-1,98	63,66	31,68	-	Hγ= 2,04-1,99<br>Hδ= 3,71-3,65	Cγ= 27,43<br>Cδ= 50,81
**Glu**	4,22	2,16-2,86	56,76	29,59	8,85	Hγ= 2,23	Cγ=35,98
**Ala**	4,27	1,35	52,83	18,88	8,02		
**D-Lys**	4,15	1,80-1,76	56,90	32,28	7,87	Hγ= 1,37<br>Hδ= 1,65<br>HƐ= 2,97	Cγ= 24,58<br>Cδ= 29,04<br>CƐ= 42,05
**Asp**	4,43	2,50-2,15	55,22	40,16	8,81		

NOE distance restraints used for structure calculation

PD-*i*3

```
assign (resid 1 and name HN) (resid 3 and name HN) 2.5 0.7 3.0
assign (resid 5 and name HN) (resid 7 and name HN) 4.0 2.2 2.0
assign (resid 10 and name HN) (resid 8 and name HN) 3.0 1.2 2.0
assign (resid 7 and name HN) (resid 9 and name HN) 4.0 2.2 2.0

assign (resid 6 and name HA) (resid 5 and name HD+) 3.0 1.2 2.0
assign (resid 10 and name HD+) resid 7 and name HA) 4.0 2.2 2.0
assign (resid 10 and name HN) (resid 8 and name HB) 4.0 2.2 2.0
assign (resid 10 and name HN) (resid 9 and name HB+) 3.0 1.2 2.0

assign (resid 1 and name HN) (resid 10 and name HB+) 3.0 1.2 2.0

assign (resid 8 and name HN) (resid 7 and name HB+) 2.5 0.7 2.0
assign (resid 9 and name HN) (resid 8 and name HG+) 3.0 1.2 2.0

assign (resid 5 and name HD+) (resid 8 and name HB) 4.0 2.2 2.0
assign (resid 5 and name HD+) (resid 6 and name HA) 2.5 0.7 2.0
assign (resid 5 and name HD+) (resid 6 and name HG+) 4.0 2.2 2.0


assign (resid 10 and name HD+) (resid 9 and name HB+) 3.0 1.2 2.0
assign (resid 10 and name HD+) (resid 7 and name HA) 3.0 1.2 2.0
```

PD-*i*6

```
assign (resid 3 and name HA) (resid 2 and name HN) 3.0 1.2 2.0
assign (resid 4 and name HA) (resid 5 and name HN) 2.5 0.7 2.0
assign (resid 5 and name HA) (resid 6 and name HN) 3.0 1.2 2.0
assign (resid 6 and name HA) (resid 7 and name HN) 3.0 1.2 2.0
assign (resid 7 and name HA) (resid 8 and name HN) 2.5 0.7 2.0

assign (resid 1 and name HA) (resid 3 and name HN) 2.5 0.7 2.0

assign (resid 1 and name HA) (resid 2 and name HD1) 4.0 2.2 2.0
assign (resid 1 and name HE3) (resid 3 and name HN) 4.0 2.2 2.0
assign (resid 1 and name HA) (resid 6 and name HB+) 3.0 1.2 2.0
assign (resid 3 and name HN) (resid 1 and name HB+) 3.0 1.2 2.0
assign (resid 1 and name HA) (resid 3 and name HG++) 4.0 2.2 2.0

assign (resid 2 and name HD1) (resid 1 and name HN) 4.0 2.2 2.0
assign (resid 2 and name HB+) (resid 6 and name HB+) 4.0 2.2 2.0
assign (resid 2 and name HA) (resid 6 and name HB+) 3.0 1.2 2.0

assign (resid 4 and name HD+) (resid 3 and name HA) 2.5 0.7 2.0
assign (resid 4 and name HA) (resid 3 and name HG++) 4.0 2.2 2.0
assign (resid 5 and name HB+) (resid 7 and name HB+) 2.5 0.7 2.0
assign (resid 5 and name HB+) (resid 4 and name HG+) 2.5 0.7 2.0
assign (resid 6 and name HA) (resid 5 and name HB+) 3.0 1.2 2.0
assign (resid 7 and name HA) (resid 8 and name HB+) 4.0 2.2 2.0

assign (resid 3 and name O) (resid 6 and name HN) 2.3 0.8 0.2
assign (resid 3 and name O) (resid 6 and name N) 3.3 0.8 0.2
```

Bibliography

(1) Bhardwaj, G.; Mulligan, V. K.; Bahl, C. D.; Gilmore, J. M. Accurate de Novo Design of Hyperstable Constrained Peptides. *Nature* **2016**, *538* (7625), 329–335. https://doi.org/10.1038/nature19791.

(2) Zak, K. M.; Kitel, R.; Przetocka, S.; Golik, P.; Guzik, K.; Musielak, B.; Dömling, A.; Dubin, G.; Holak, T. A. Structure of the Complex of Human Programmed Death 1, PD-1, and Its Ligand PD-L1. *Structure* **2015**, *23* (12), 2341–2348. https://doi.org/10.1016/j.str.2015.09.010.

(3) Cheng, X.; Veverka, V.; Radhakrishnan, A.; Waters, L. C.; Muskett, F. W.; Morgan, S. H.; Huo, J.; Yu, C.; Evans, E. J.; Leslie, A. J.; et al. Structure and Interactions of the Human Programmed Cell Death 1 Receptor. *Journal of Biological Chemistry* **2013**, *288* (17), 11771–11785. https://doi.org/10.1074/jbc.M112.448126.

(4) Wishart, D. S.; Bigam, C. G.; Holm, A.; Hodges, R. S.; Sykes, B. D. 1H, 13C and 15N Random Coil NMR Chemical Shifts of the Common Amino Acids. I. Investigations of Nearest-Neighbor Effects. *Journal of Biomolecular NMR* **1995**, *5* (1), 67–81. https://doi.org/10.1007/BF00227471.

(5) Schwieters, C. D.; Bermejo, G. A.; Clore, G. M. Xplor-NIH for Molecular Structure Determination from NMR and Other Data Sources. *Protein Science* **2018**, *27* (1), 26–40. https://doi.org/10.1002/pro.3248.

(6) Cierpicki, T.; Otlewski, J. Amide Proton Temperature Coefficients as Hydrogen Bond Indicators in Proteins. *Journal of biomolecular NMR* **2001**, *21* (3), 249–261. https://doi.org/10.1023/a:1012911329730.

(7) Kortemme, T.; Kim, D. E.; Baker, D. Computational Alanine Scanning of Protein-Protein Interfaces. *Science's STKE: signal transduction knowledge environment* **2004**, *2004* (219). https://doi.org/10.1126/stke.2192004pl2.

(8) Buchan, D. W. A.; Jones, D. T. The PSIPRED Protein Analysis Workbench: 20 Years On. *Nucleic Acids Research* **2019**, *47* (W1), W402–W407. https://doi.org/10.1093/nar/gkz297.

(9) Kozakov, D.; Grove, L. E.; Hall, D. R.; Bohnuud, T.; Mottarella, S. E.; Luo, L.; Xia, B.; Beglov, D.; Vajda, S. The FTMap Family of Web Servers for Determining and Characterizing Ligand-Binding Hot Spots of Proteins. *Nature Protocols* **2015**, *10* (5), 733–755. https://doi.org/10.1038/nprot.2015.043.
